# Innovative Bioactive Nanofibrous Materials Combining Medicinal and Aromatic Plant Extracts and Electrospinning Method

**DOI:** 10.3390/membranes13100840

**Published:** 2023-10-21

**Authors:** Nikoleta Stoyanova, Nasko Nachev, Mariya Spasova

**Affiliations:** Laboratory of Bioactive Polymers (LBAP), Institute of Polymers, Bulgarian Academy of Sciences, Acad. G. Bonchev St., bl. 103A, BG-1113 Sofia, Bulgaria; nstoyanova@polymer.bas.bg (N.S.); nachev_n@polymer.bas.bg (N.N.)

**Keywords:** medicinal and aromatic plant extracts, electrospinning, bioactive nanofibrous materials, treatment of human diseases

## Abstract

Since antiquity, humans have known about plants as a medicinal cure. Recently, plant extracts are attracting more attention as a result of their natural origin and wide range of desirable features. Nanotechnology’s progress and innovations enable the production of novel materials with enhanced properties for a broad range of applications. Electrospinning is a cutting-edge, flexible and economical technique that allows the creation of continuous nano- and microfibrous membranes with tunable structure, characteristics and functionalities. Electrospun fibrous materials are used in drug delivery, tissue engineering, wound healing, cosmetics, food packaging, agriculture and other fields due to their useful properties such as a large surface area to volume ratio and high porosity with small pore size. By encapsulating plant extracts in a suitable polymer matrix, electrospinning can increase the medicinal potential of these extracts, thus improving their bioavailability and maintaining the required concentration of bioactive compounds at the target site. Moreover, the created hybrid fibrous materials could possess antimicrobial, antifungal, antitumor, anti-inflammatory and antioxidant properties that make the obtained structures attractive for biomedical and pharmaceutical applications. This review summarizes the known approaches that have been applied to fabricate fibrous materials loaded with diverse plant extracts by electrospinning. Some potential applications of the extract-containing micro- and nanofibers such as wound dressings, drug delivery systems, scaffolds for tissue engineering and active food packaging systems are discussed.

## 1. Introduction

Since ancient times, mankind has had an intense interest in different therapeutic strategies based on plant-derived medications for the treatment and prevention of some major diseases [1]. The therapeutic benefits of plants are of considerable interest to both the educated public and the medical community, but there is much uncertainty regarding their identification, efficacy, therapeutic dose, toxicity, standardization and regulation. Nowadays, the utilization rate of plant extracts in feed additives, as resources of medicinal compounds, increases progressively, aiming to cope with the final customers’ demands for high-quality natural and safe products.

In recent years the creation of micro- and nanofibrous structures is an area that has been the subject of increasing scientific interest. This is mainly because these materials consist of fibers with diameters in the nano- and microscale range possessing large specific surface area and a small pore size which renders these materials suitable for application in medicine, pharmacy, food packaging, cosmetics, agriculture, electronics, etc.

Methods such as drawing, template method, phase separation, self-assembly, extrusion and electrospinning are used to fabricate micro- and nanofibers [2]. Among them, electrospinning is a versatile, cost-effective and efficient method used to obtain micro- and nanofibrous structures with a desired morphology and composition [3]. The electrospun fibers could find applications in biomedicine, such as in drug delivery systems, for tissue engineering, or for wound healing; in cosmetics; in food and food packaging applications; or in agriculture. Moreover, they could be applied as functional materials and devices such as composite reinforcement, fibrous filters, protective clothing, smart textiles and in energy and electronics such as batteries/cells and capacitors, sensors and catalysts [4,5,6,7,8].

The creation of suitable carriers for encapsulation of plant extracts is a prerequisite for their future applications and involves the proper choice of carrier matrix and technique for preserving extract biological activity while ensuring high encapsulation efficiency and an effective delivery process. Polymers played an important role in the development of carriers for various biologically active substances (BAS) allowing diverse ways of delivering dosages over extended periods of time for hydrophilic as well as for hydrophobic BAS while being chemically inert and relatively low cost [9]. Moreover, the use of polymer carriers for drug delivery has grown due to the ability to easily tune their physicochemical and biological properties [10].

In the last years, due to innovative formulation techniques, there is an increasing interest in the fabrication of non-spherical carriers with elongated or filamentous morphologies. These supramolecular structures’ distinctive physiochemical characteristics provide distinct benefits as drug delivery systems. Recently, electrospinning is a versatile, flexible and efficient method for the encapsulation of BAS into fibrous materials allowing the creation of highly efficient delivery systems [11]. Due to their porosity, electrospun fibers can incorporate active substances in their volume or immobilize biologically active molecules on their surface by chemical or physical adsorption [12]. By adjusting the solution and process parameters as well as the environmental conditions, the fibrous structure, morphology and porosity can be controlled.

## 2. Plant Extracts (Ple) as Medicinal Cures for Prevalent Diseases

Increased oxidative stress and inflammatory conditions are linked to a wide range of disorders, including diabetes, cancer, infections, atherosclerosis, cardiovascular diseases, Alzheimer’s disease and other degenerative diseases [13,14,15].

The above-mentioned diseases are among the most prevalent diseases affecting people, and some of them are currently ranked as a leading cause of death in the world. The dreadful disease known as cancer is multifactorial and genetically complex, and no treatment is 100% effective. Globally, it has increased the economic burden and become a significant social concern [16]. Chronic kidney diseases as well as chronic respiratory diseases are among the other widespread diseases [17]. Prevention and successful treatment of these prevalent diseases are extremely important for the future health and quality of life of people as well as for the global economy and the improvement of healthcare management. Thousands of years ago, fragrant plants and herbs, as well as their extracts and essential oils, were used in traditional medication, veterinary medicine, pharmacy, cosmetics, the food processing industry as well as for agricultural use. The World Health Organization has recognized the successful application of more than 20,000 plants for therapeutic purposes [18]. Table S1 (Supplementary Material) summarizes the benefits of some of the most used plant extracts for health.

**Figure 1:**
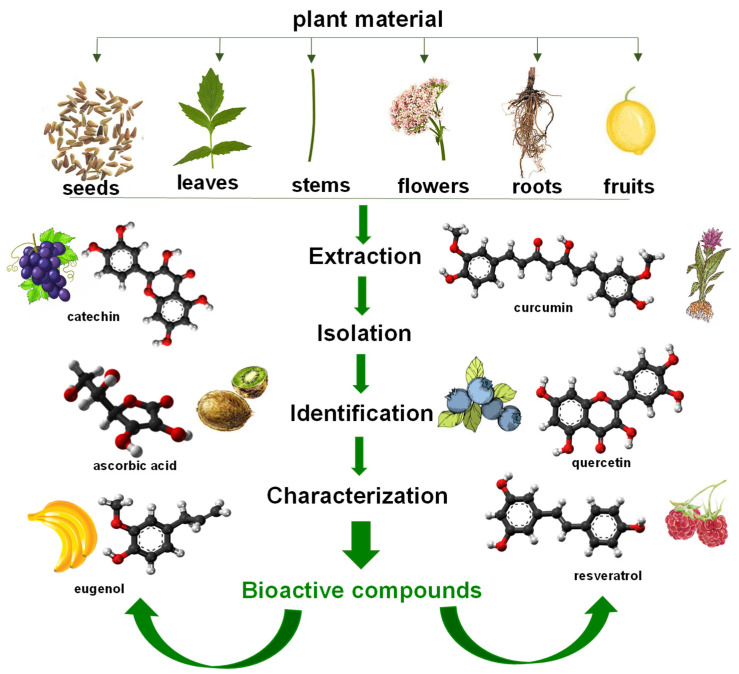
Schematic representation of general steps for extraction of bioactive compounds from plant materials.

The schematic representation of general steps for the extraction of bioactive compounds from plant materials is shown in Figure 1. The process of extracting various parts of medicinal plants involves separating active plant components or secondary metabolites, including flavonoids, alkaloids, saponins, terpenes, steroids and glycosides from inert or inactive components, using the right solvent and accepted extraction techniques. Various techniques were employed to extract therapeutic herbs, including infusion, maceration, digestion, percolation, decoction and Soxhlet extraction, superficial extraction, and ultrasound-assisted and microwave-assisted extraction [19,20,21,22]. Secondary metabolites can also be isolated and purified using thin-layer chromatography, high-performance liquid chromatography, paper chromatography and gas chromatography [23,24]. The suitable extraction method is determined by the type of plant material, the solvent used, the pH of the solvent, the temperature, and the solvent to sample ratio, as well as the uses for which the plant extract is intended.

In order to cure age-related disorders including Alzheimer’s disease, Parkinsonism, anxiety and depression, plant extracts such as *Achyrocline tomentosa*, *Eupatorium viscidum*, *Ruprechtia apetala*, *Trichocline reptans*, *Zanthoxylum coco*, *Poncirus trifoliate*, *Treculia obovoidea*, *Angelica archangelica*, *Cassia obtisufolia*, *Desmodium gangeticum*, *Salvia officinalis*, *Moringa oleifera*, *Ginkgo biloba*, *Lavandula officinalis* and *Prunus armeniaca* L. with high concentrations of phenolic compounds and flavonoids were widely used as a successful treatment of the abovementioned diseases [25,26,27]. Furthermore, evidence-based pharmacological studies demonstrate that flavonoids have a significant role in the prevention and management of chronic kidney disease as well as renal fibrosis. Some flavonoids isolated from plant extracts (Hesperidin, Quercetin, Rutin, Luteolin, Morin, etc.) can prevent renal dysfunction and enhance renal function by blocking or reducing harmful processes such as oxidative stress and inflammation [28]. *Rheum* spp., *Astragalus membranaceus*, *Cordyceps sinensis*, *Triptirygium wildfordii Hook F*, *Abelmoschus manihot*, *Salvia miltiorrhiza*, *Vitis vinifera*, *Zingiber officinale* and *Garcinia lucida* extracts are used to treat renal diseases as well [29,30].

One of the common causes of death worldwide is cancer, and despite advances in the development of the drug industry, there is a huge need to develop novel plant-derived medications for the treatment and prevention of this insidious disease. Natural herbal medicines offer several benefits over traditional chemical drugs in order to reduce the chemotherapy negative effects. A number of medicinal plant extracts (*Portulaca oleracea*, *Melissa Officinalis*, *Curcuma longa*, *Allium sativum*, *Rheum Palmatum*, *Salvia officinalis*, *Geranium robertianum*, *Ginkgo biloba* L., *Rosmarinus officinalis*, *Calendula officinalis*, etc.), have been shown to have anti-cancer action on various human cancer cell lines [31,32,33].

Diabetes mellitus is a metabolic disorder characterized by abnormally increased blood glucose levels. Due to the negative effects associated with oral hypoglycemic drugs for the treatment of diabetes mellitus, there is an increasing interest in herbal therapies. As a result, traditional herbal treatments derived from plants are mostly employed, and they play an essential role in the management of this disorder. For instance, several studies have confirmed the anti-diabetic properties of *Acacia nilotica*. Its hypoglycemic effects have been attributed to its role in stimulating the islets of Langerhans to produce more insulin resulting in reduced blood sugar [34]. The presence of phenols, flavonoids, alkaloids, tannins, phylobatanins and saponins in these plant extracts may be responsible for their hypoglycemic effects [35]. *Berberis vulgaris* L. is a popular plant that has long been used medicinally and nutritionally in Europe, Asia and America. Especially, the root bark and stems of *B. vulgaris* have therapeutic benefits due to berberine, an isoquinoline alkaloid found in these plant parts. Berberine is widely recognized for its anti-diabetic properties. It reduces blood glucose levels in both healthy and diabetic patients and promotes insulin secretion in both groups. Furthermore, it may lower fasting and postprandial blood glucose, food and water consumption, as well as boosting the anti-diabetic benefits of other medications such as canagliflozin [36].

Furthermore, *Ginkgo biloba* L. leaf extract is believed to provide several health benefits for the brain, heart, and blood vessels as well as anti-inflammatory, anti-apoptotic, and platelet-activating factor properties. The plant-based remedies have also been utilized to treat ischemic heart disease, hypertension, arteriosclerosis, thrombus formation, and more recently, diabetes mellitus prevention. *Ginkgo biloba* contains various biologically active compounds that affect insulin action and production [37]. Moreover, many phenolic and flavonoid compounds, such as rosmarinic acid (RA), ferulic acid (FA), caffeic acid (CA), betulinic acid (BA), quercetin, rutin, etc., have been found in the medicinal plant *Melissa officinalis* (*M. officinalis*, lemon balm), and it possesses antioxidant, antidiabetic, antiproliferative, cytotoxic, antimicrobial and hypotensive properties [38]. It has been proven that *M. officinalis* had benefits in treating hyperlipidemia, hypertension and glycemic status [39].

Phytocannabinoids, which influence a variety of epilepsy-related targets, are among the more than 300 substances (lipophilic phytocannabinoids) present in *Cannabis sativa* L. and have varying therapeutic potentials. It has been demonstrated that components in cannabis, particularly cannabinoids, interact with the targets (CNR1, ALB, GSK3BA, AR and MAPK10) to alleviate epilepsy. Additionally, a molecular docking investigation supported cannabis’ ability to treat epilepsy [40]. In addition, *Portulaca oleracea* (*P. oleracea*) and its active ingredients have been demonstrated to have significant neuroprotective, neuroregenerative and antinociceptive activities [41].

In fact, numerous powerful medications for a variety of human conditions, including respiratory illnesses, have been made from plant extracts. *Curcuma longa (C. longa)*, commonly known as turmeric, is a perennial plant in the *Zingiberaceae* (ginger) family. This plant is employed as a primary ingredient in food preparation and is classified as a functional food because of its possible health advantages. This plant has long been used to treat inflammatory diseases, menstrual problems, hematuria, hemorrhage, colic, urticarial, skin allergy, viral hepatitis, inflammatory joint conditions, sore throat, wounds, anorexia, jaundice, liver disorders, rheumatism, runny nose, cough, diabetic wounds, sinusitis, as well as asthma and allergies [42]. The therapeutic benefits of *C. longa* and its components, primarily curcumin, on asthma, lung cancer, and respiratory bacterial and viral infections have been demonstrated as well [43].

Being a member of the *Alliaceae* family, garlic, also known as *Allium sativum* L., has long been recognized as a plant able to treat a variety of diseases and physiological conditions. Allicin is the substance that makes garlic active. It is well known that this herb possesses anti-diabetic, anti-bacterial, and anti-cancer activities along with anticoagulant, anti-inflammatory, immunomodulatory, antioxidant, hepatoprotective and wound healing effects [44].

It is known that *Punica granatum* (pomegranate) extract contains bioactive molecules that are effective in the treatment of oral cavity diseases, and particularly, of the oral cavity in patients with HIV/AIDS. According to the existing literature, the principal chemicals identified through phytochemical analysis include phenolics and flavone glycosides, triterpenoids and hydrolyzable tannins [45]. Moreover, *Punica granatum* has beneficial effects in rheumatoid arthritis treatment [46].

*Achyranthes aspera* L. (family *Amaranthaceae*) is a valuable medicinal plant used for the treatment of asthma, dysentery, hypertension, malarial fever and diabetes. *Rosa centifolia* L. is a perennial plant that is usually referred to as the hundred-leaved rose. In traditional medicine, it is used to treat inflammatory diseases such as arthritis, asthma, cough, wounds, bronchitis and ulcers. Flavonoids are abundant in *Rosa centifolia* extract [47]. In conventional medicine, *Rosa centifolia* L. has been used to treat rheumatoid arthritis and joint discomfort [48].

In the past decades the use of medicinal and aromatic plant extracts and related products continue to increase exponentially. This is due to the fact that these extracts and extract-based products possessed complex biological activities able to treat diverse symptoms of various human diseases. Therefore, many people around the world rely on biologically active extract-based medicines as a major health care source. However, there is growing demand for suitable carriers that will enhance the therapeutic potential of plant extracts.

## 3. Biopolymers and Natural Polymers Used as Carriers of Plant Extracts

Biopolymers are macromolecules, produced from natural sources such as bacteria, fungus, plants, and are even chemically synthesized by people from biological materials such as maize, starch, cellulose, agarose, pullulan, carrageenan, chitin and corn. Due to their biological origin, biopolymers are ideal alternative resources that are continually being explored to minimize problems associated with environmental problems. Because of their biocompatibility and/or biodegradability, biopolymers are useful in a variety of applications, including edible films, emulsions, packaging materials in the food industry, drug transport materials, medical implants, wound healing, tissue scaffolds, dressing materials, etc. [49]. Although it relies on their source, type, and production process, choosing the right biopolymers for different purposes is crucial. Numerous biopolymers combined with nanotechnology, may be used to provide a wide range of material applications for water purification, in biomedicine, energy generation, food production, cosmetic surgery, 3D printing, drug delivery and tissue engineering [50].

Depending on their origin and method of production, the biopolymers could be divided into three main groups:

(1) Polymers that were directly extracted or separated from biomass, like starch, cellulose, arabinoxylan, and lignin;
(2) Polymers that were synthesized from bio-derived monomers like polylactic acid (PLA) and cellulose acetate (CA);
(3) Polymers that were produced by microorganisms such as polyhydroxyalkanoates (PHAs) and polysaccharides [51].

**Figure 2:**
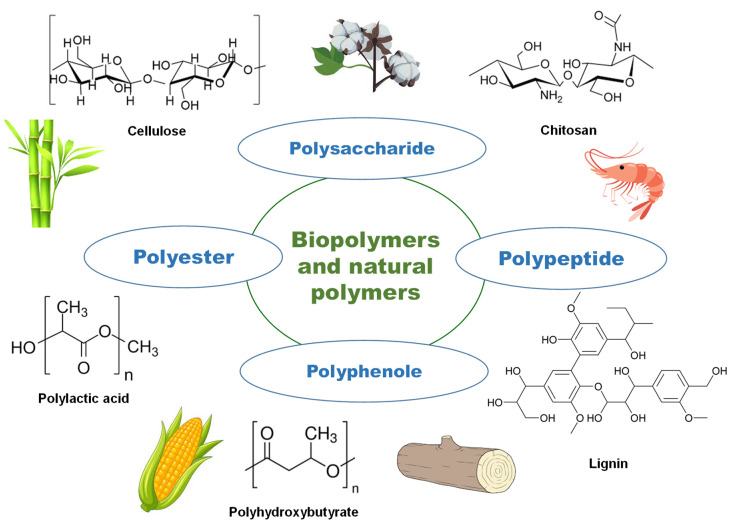
Structure of the most abundant biopolymers and natural polymers.

The structures of the most abundant biopolymers and natural polymers are shown in Figure 2.

A variety of biopolymers have often been employed as biopolymer-based carriers for plant extracts. The characteristics of the plant extract and the intended application of the resulting material determine the appropriate biopolymer and encapsulation technique selection. Biopolymer-based encapsulation is a promising technique for increasing the functional qualities of natural substances in food and beverage products and has potential application in the pharmaceutical and cosmetic sectors. Encapsulation of plant extracts into polymer-based formulations, for example, micelles, micro- and nanoparticles, films, micro-and nanofibers or hydrogels, is provided to ensure decreasing volatility, improved stability and water solubility. For instance, a bioactive plant extract (*Euclea schimperi*) derived from one of Oman’s native plants was loaded into bacterial cellulose (BC) by using an ex situ composite development approach. This way, composite materials for potential biomedical applications were fabricated. BC had a significant potential for extract-holding capacity, which gave it bactericidal effects against Gram-positive bacteria *Staphylococcus aureus* (*S. aureus*). In addition, the bactericidal impact of the composite revealed that bioactive chemicals in the plant extract preserved their bactericidal properties. The development of bacterial cellulose composites with other plant extracts reveals their therapeutic value in the development of medical, cosmetic and pharmaceutical goods [52]. On the other hand, biodegradable films have the potential to replace many of today’s widely used packaging materials, which are based on petroleum polymers [53]. Hassanloofard et al. demonstrated the potential for creating biodegradable films from cellulose acetate combined with the extract from *Falcaria vulgaris* (*F. vulgaris)* using the casting process. The results revealed that when the amount of *F. vulgaris* extract was increased, the tensile strength and modulus of elasticity of the fabricated CA films decreased. Due to the extract’s hydrophobic properties, the increase in concentration resulted in a reduction in vapor permeability, water absorption, swelling percentage and water content. The presence of phenolic compounds in CA film enhanced the antioxidant activity. CA films loaded with the extract showed greater oxygen permeability than the neat CA. The results demonstrated that active films containing various concentrations of *F. vulgaris* extract exhibited effective antibacterial activity against *Candida albicans (C. albicans)*, *S. aureus*, *Escherichia coli (E. coli)* and *Candida glabrata*. This reveals the potential of the obtained films to find application as food preservatives [54]. Furthermore, Gradinaru et al. fabricated chitosan membranes containing two medicinal plant extracts (*Hypericum perforatum* and *Salvia officinalis*) with antimicrobial activity. The incorporation of the plant extracts enhanced the antibacterial properties of the resulting films against *S. aureus*, *E. coli* and *Pseudomonas aeruginosa*. The obtained results show that the prepared chitosan-based films are promising candidates that could be used as wound dressing material due to their good physico-chemical properties and antimicrobial activity [55]. Additionally, microencapsulation in alginate beads was used to protect the functional properties of *Lamiaceae* bioactives (lavender, lemon balm, peppermint, sage, and thyme) [56]. *Syzygium cumini*, *Bauhinia variegata*, *Cedrus deodara*, *Lonicera japonica* and *Eleaocarpus sphaericus* are a few examples of medicinally significant plants whose leaves have been used to create stable polylactid acid (PLA)-based nanoparticles (NPs) by Kumari et al. The authors have effectively encapsulated quercetin, on a most consistently distributed PLA-based NP prepared using *Lonicera japonica* leaf extract to investigate such NPs for drug/small molecule delivery. A gradual and persistent release of the quercetin molecule from NPs was observed. This environmentally friendly method, which relies on the stable PLA NPs produced by the use of plant extracts, opens the door to the encapsulation of drugs, small molecules and other bioactive components for improved cellular absorption, biodistribution and targeted delivery. From this point of view, PLA NPs might be helpful to increase the therapeutic efficacy of small molecules/drugs [57].

Moreover, the well-known medicinal herbs *Pistacia lentiscus* and *Calendula officinalis* (*C. officinalis*) were incorporated in highly linked 3D scaffolds from skin collagen. The collagen-based materials demonstrated a low cytotoxic effect and good biocompatibility with skin cells such as keratinocytes and fibroblasts. Moreover, the plant extracts loaded into collagen may accelerate the re-epithelialization of patients and prevent infections. Therefore, the fabricated scaffolds might be employed as suitable wound dressing materials [58].

There are data in the literature showing the successful incorporation of ethanolic extracts of *Rosmarinus officinalis* aerial parts, *Achillea millefolium* and *C. officinalis* flowers in carbomer-based hydrogel dressings [59]. The obtained results reveal that the prepared hydrogel formulations could find potential applications as innovative materials for wound treatment in biomedicine. The presented studies reveal the way to develop really eco-friendly green materials is by combining medicinal plant extracts with the suitable biodegradable matrix in order to create novel biomaterials with desired and tailored properties for diverse applications.

## 4. Electrospinning Method

An increasing number of scientific studies have recently focused on the production of micro- and nanofibrous materials. There are several basic methods for obtaining polymer micro- and nanofibers including the method of self-organization [60], the template method [61], by phase separation [62], drawing [63], blowing (extruding) from melt [64] as well as by electrospinning [65,66]. Among these methods electrospinning is the only method that is relatively easy, efficient and enables the production of fibrous materials of the desired design and morphology [65,67,68]. In recent years, electrospinning has been one of the most dynamic developing nanotechnologies worldwide. Moreover, it is extremely attractive due to the possibility of the industrial production of non-woven fabrics. The two main processes differ depending on the solution viscosity: electrospraying or electrospinning [69]. When dilute polymer solutions are used, electrospraying occurs resulting in micro- or nano-sized particle production. A fiber fabrication process begins when concentrated polymer solutions are subjected to an electric field. This process is known as electrospinning, and it allows the production of irregularly deposited or oriented fibers, possessing remarkable properties, with diverse morphology, large specific surface area and porous structure. Fibrous mats can be obtained from solution or melt and may find potential application in medicine (mats with antitumor, antibacterial or hemostatic activity), in the pharmacy (drug carriers), filtration materials or photocatalytic removal of organic pollutants in the purification of water, for the immobilization of enzymes or for the preparation of fibrous materials with magnetic properties [12,70,71,72,73].

### 4.1. Schematic Representation of the Electrospinning/Electrospraying Set-Up

**Figure 3:**
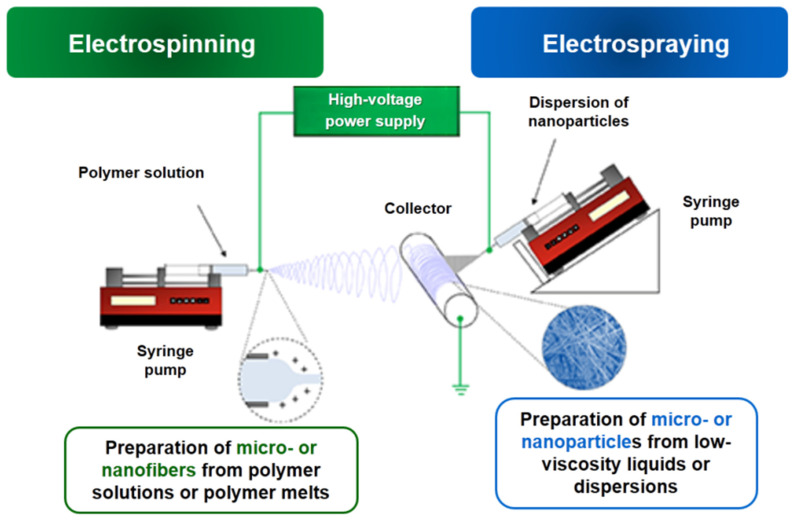
Schematic representation of the electrospinning/electrospraying set-up.

The schematic representation of the typical electrospinning/electrospraying set-up is shown in Figure 3. The equipment for this process consists of three basic elements: a high-voltage power supply, a reservoir with a capillary (needle) where the polymer solution is placed and a metal collector (static or rotating) on which the fibers/particles are deposited (Figure 3). The presence of pumps allows the solutions to be fed at a controlled flow rate. Moreover, the jet trajectory can be controlled through the use of additional focusing devices. Once a voltage is applied, the electrostatic forces deform the droplet formed at the end of the capillary/needle into a cone shape called a Taylor cone. When the electrostatic forces overcome the surface tension, a jet is ejected from the tip of the cone. Initially, the jet moves in a straight line, but after a while it begins to perform whip-like movements due to the high charge density causing extensive plastic deformation. Meanwhile, during the jet’s flight the solvent evaporates, and dry nano- and microfibers are deposited on the collector.

### 4.2. Factors Influencing the Process

The formation of fibers by electrospinning is a process that is influenced by many factors, including the parameters of both the spinning solution and the electrospinning process. The polymer solution must have a sufficiently high viscosity for the conduction of electrospinning. Lower viscosity values result in preparation of defective fibers, while higher values result in cylindrical fibers with a larger diameter [74]. It is well known that electrospinning occurs when, under the action of electrostatic forces of the applied field from a polymer solution or melt in the presence of a sufficient number of chain entanglements, an electrically charged jet is drawn, leading to the formation of fibers. The following equation can be used to calculate the number of chain entanglements during the process of fiber formation.

(1) $${\left(n_{e}\right)}_{soln}=\frac{{\bar{M}}_{w}}{{{(\bar{M}}_{e})}_{soln}}=\frac{\varphi_{\rho }{\bar{M}}_{w}}{{\bar{M}}_{e}}$$

where *M_w_*—weight average molar mass, *φ_p_*—volume fraction of the polymer in the solution, and *M_e_*—molar mass of chain entanglement in melt [67]. The authors observed that electrospinning produced fibers with defects at values of (*n_e_*)*_soln_* = 2 and defect-free fibers at (*n_e_*)*_soln_* > 3.5. The analysis performed by Shenoy and co-authors showed that fibers begin to form in the presence of chain entanglements. The validity of Equation (1) has been confirmed for solutions of polystyrene, poly (lactic acid), polyethylene oxide (PEO) and polyvinylpyrrolidone (PVP). It should be noted that this model is only valid for polymer solutions dissolved in good solvents, and in some cases deviations from Shenoy’s theory have been observed when working with highly volatile solvents.

**Figure 4:**
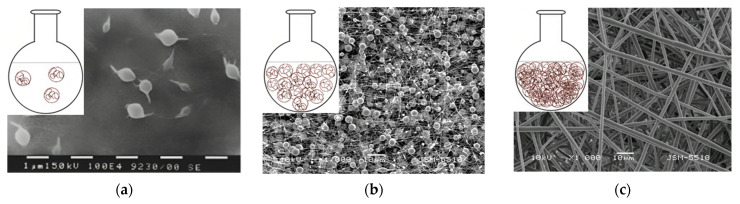
Schematic representation of polymer chains at different concentration regimes and SEM micrographs obtained from solutions with different concentrations: (**a**) dilute solution, (**b**) semi-dilute without entanglement between polymer chains and (**c**) semi-dilute with entanglement between polymer chains.

Depending on the concentration, the polymer solutions could be divided into dilute, semi-dilute without entanglement of the polymer chains, semi-diluted with entanglement of the polymer chains and concentrated. A schematic representation of the modes is shown in Figure 4 (inset figures). SEM micrographs of particles, fibers with defects, and defect-free fibers obtained at the different concentration regimes are shown as well. In dilute solutions, the polymer chains interact mainly with the solvent molecules (Figure 4a). In semi-dilute solutions without chain entanglement (Figure 4b), the concentration has not reached the required level for an optimal number of entanglements between the polymer chains. In semi-diluted solutions with entanglement of the polymer chains, a certain concentration is reached, which is necessary for sufficient entanglements. Typically, the number of entanglements in the solution must be above two to initiate fiber formation (Figure 4c) [75,76].

It is well known that the solution concentration and viscosity are strongly correlated. As the viscosity or concentration of the solution increases, the distribution of mean fiber diameters becomes more uniform [77]. The only objects produced are spheres or fibers with spherical defects by applying a high voltage to polymer solutions with low viscosity. However, above a certain critical concentration, a continuous fibrous structure is formed, and the concentration of the solution has a crucial impact on the fibrous morphology [78].

The electrical conductivity of the solution is another factor that influences the diameter of the obtained fibers. It depends on the polymer type, the solvent used and the presence of ionized salts. Salts added to polymer solutions increase the charge density of the jet, leading to repulsion between individual charges, thereby increasing the extent of jet elongation and forming fibers with smaller diameters [79].

The surface tension of the spinning solution also has an effect on the morphology of the resulting fibers. For the electrospinning process to take place, the solution must overcome the surface tension. It may depend on the type of solvent used. Generally, higher solution surface tension values hinder the electrospinning process and lead to the formation of particles or fibers with defects [80].

The applied voltage is a critical process factor. Typically, a positive or negative voltage of over 6 kV is required for the jet to form a Taylor cone. When the voltage is low, the electrostatic forces cannot overcome the solution drop’s surface tension, and as a result, a jet is not drawn, but a drip occurs [81]. As the applied voltage increases, the electrostatic forces also increase, which leads to the formation of a jet and the process of electrospinning begins [82]. Some authors believe that increasing the applied voltage leads to the creation of a stronger electric field. This, consecutively, contributes to the production of thinner fibers [83]. Other researchers contend that the strong electric field increases the jet’s acceleration and shortens its flight time. A longer jet flight time means more time for the fibers to stretch and elongate before being deposited on the collector. Thus, the lower voltage, reduced acceleration and weaker electric field can increase the flight time of the jet, resulting in the formation of finer fibers.

The distance between the capillary tip and the collector, the flow rate of the spinning solution, the rotation speed of the collector and the type of collector, are all electrospinning process parameters that influence the morphology of the fibers as well. Above a certain critical value of solution flow rates, uniform fibers with no defects are produced. An increase in the rate above the critical value leads to particle formation [84].

The time of flight for the jet is found to be shortened when the distance between the tip of the capillary and the collector surface is decreased. If the distance is too short, there is insufficient time for the solvent to evaporate, and the fibers do not dry before they reach the collector. Subsequently, the solvent-containing fibers are deposited [85]. Some authors reported that a larger distance favors the formation of thinner fibers [86]. It is also known that solutions of water-soluble polymers require the use of a greater distance for the formation of dry fibers compared to systems using volatile organic solvents [68].

Environmental factors such as temperature and humidity have a significant impact on morphology, diameter and diameter distribution of the electrospun fibers. It was observed that when the temperature rises, thinner fibers are produced. This is because high temperatures cause the polymer solutions’ viscosity to decrease [87]. The predominant number of studies on the electrospinning of polymer solutions were performed in air environment conditions. The formation of thicker fibers or fibers with defects at higher relative humidity is detected which is most probably due to the fact that a greater discharge of electrostatic charges from the surface of the polymer solution occurs, causing a decrease in the forces of the electrical charges [88].

The use of various collectors and focusing devices can alter the trajectory of the jet and change the morphology and orientation of the prepared fibers. A stationary metal plate or foil, positioned at a certain distance from the needle, serves as the most basic collector in electrospinning. Fibers are typically deposited randomly on the metal plate when using this type of collector [89]. The use of a drum or disk collector has the advantage of producing oriented fibers and long oriented bundles [90]. However, one disadvantage of their use is that the fiber orientation decreases with layer thickness. Collectors that are wire-wrapped, wire-built or bladed can also be used to create highly oriented fibers [91].

The electrospinning method provides the possibility for creation of novel nanofibrous architectures possessing a complex of desired properties. As it was presented, many diverse parameters influence this electrohydrodynamic process. Therefore, the fabrication of these nanostructures requires knowing and being able to control the solution and process parameters. The addition of bioactive compounds such as medicinal and aromatic plant extracts impacts the spinnability of the solution and therefore is a crucial factor in determining the morphology, structure and properties of the obtained fibrous materials as well.

## 5. Plant Extracts Incorporated by Electrospinning

In the last several years there has been a great interest in the fabrication of nanofibrous materials containing bioactive plant extracts by electrospinning. Up to now, the numbers of publications concerning the topic of fabrication of nanofibrous materials containing plant extract by electrospinning are much fewer compared to publications discussing the preparation of drug-containing fibers by electrospinning. Nevertheless, the number of studies increased due to the significant advantages of the natural compounds such as inherent medicinal activity, non-toxicity, lack of side effects, environmental sustainability, cost-effectiveness and easy availability. Moreover, the created hybrid structures possess antibacterial, antifungal, anti-inflammatory, antioxidant and anticancer activities which make these fibrous materials attractive for biomedical, pharmaceutical, agricultural and industrial applications. For the most part, articles on prepared nanofibers containing plant extracts were addressed to their prospective use in medicine.

Many researchers have reported the incorporation of *C. longa* in different polymer fibrous materials by electrospinning [92]. Moreover, this medicinal plant is the first to be loaded into electrospun fibers. The first report concerning the loading of curcumin in cellulose acetate (CA) was published in 2007 [93]. For the fabrication of the hybrid mats, CA with *Mw* = 30,000 Da and a degree of acetyl substitution of ~2.4 was used. The CA solution concentration was 17 wt% in mixed solvent acetone/dimethylacetamide 2:1 *v*/*v*. The curcumin was in the amount of 5 to 20 wt%. The obtained fibers were smooth with the average diameters of the curcumin-loaded CA fibers measuring up to 340 nm. The authors have proved that electrospun curcumin-loaded CA materials are non-toxic to normal human dermal fibroblasts.

Curcumin was incorporated in CA and polyvinylpyrrolidone (PVP) fibrous materials by one-pot electrospinning or dual spinneret electrospinning enabling modulating drug release [94]. PVP assisted faster curcumin release while curcumin imparted antimicrobial properties to the novel mats rendering the created materials suitable for wound dressing applications.

**Figure 5:**
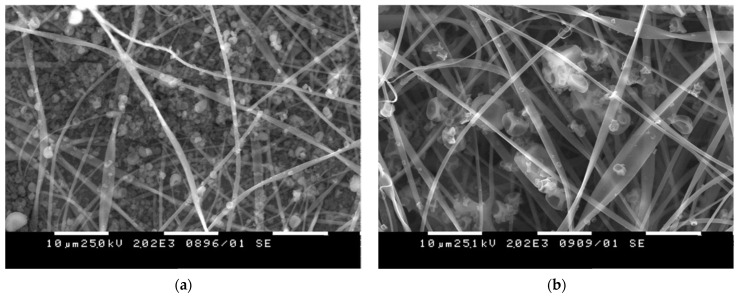
SEM micrographs of Curc/PVP-*on*-CA mats using (**a**) acetone/water and (**b**) ethanol/acetone solvent system.

Innovative materials that allow the enhanced release of curcumin in aqueous medium were obtained from CA with electrosprayed curcumin/polyvinylpyrrolidone (Curc/PVP) particles [92,94]. The use of PVP led to hydrophilization of the mats and facilitated Curc release. Different solvent systems for the dissolution of Curc/PVP were studied: acetone/water (70/30) and ethanol/acetone (50/50). The representative SEM micrographs of the prepared Curc/PVP-*on*-CA mats using different solvents were presented in Figure 5. As it can be seen, the used solvent system for the dissolution of Curc/PVP strongly influenced the particles’ morphology. The electrosprayed particles possessed a polygonal shape which is attributed to the rapid solvent evaporation. Particles with larger sizes were obtained by dissolving Curc/PVP in a more rapidly evaporated ethanol/acetone solvent system. Moreover, the authors proved that the created curcumin-containing fibrous mats possessed antibacterial activity against *E. coli* and *S. aureus* along with high in vitro cytotoxicity towards HeLa tumor cells. These features make the prepared materials promising candidates for wound dressing applications and local cancer treatment.

Curcumin was successfully loaded along with cyclodextrin (CD) in electrospun polyvinyl alcohol (PVA) nanofibers. The influence of the plant addition to the fiber morphology and structure was studied. It was proven that curcumin was presented as crystalline aggregates into the fibrous mat while preserving its chemical structure. The incorporation of curcumin in the fibers increased its thermal stability [95]. PVA was chosen as a polymer for incorporation of curcumin by electrospinning by Mahmud et. al. as well [96]. The prepared fibrous PVA/curcumin crosslinked through heat and UV treatment materials showed antibacterial efficacy against *S. aureus* and *E. coli* bacteria for biomedical applications.

A blended solution of PVA, honey and *C. longa* extract was used for the preparation of antibacterial wound dressing electrospun nanofibrous material [97]. The average diameter of the obtained nanofibers was about 340 nm and the fibers had better moisture management properties compared to nanofibrous PVA material alone. In addition, the created novel material reveals antibacterial activity. Curcumin–honey-loaded multilayered PVA/CA electrospun nanofibrous mats were obtained as well [98]. They were fabricated to serve as bioactive wound dressings. The prepared hybrid novel materials show potential resistance towards *E. coli* and ca. 90% antioxidant activity when used against diphenyl-picrylhydrazyl (DPPH) radical scavenging. In addition, the obtained material possessed excellent absorption with controlled transmission of wound exudate.

There are reports in the literature revealing the creation of nanofibers based on curcumin and PLA by electrospinning for wound healing and cancer treatment. Pankongadisak et al. have varied the amount of loaded curcumin at 0.2, 0.5 and 1.0% *w*/*w* (based on the weight of PLLA) in the PLLA solution. The developed fibers possessed a smooth surface revealing a complete curcumin incorporation. The mean diameter of the prepared fibers was between 333 and 386 nm. The higher loading of curcumin resulted in an increased release amount. It was demonstrated that the obtained fibrous materials were non-toxic to human adult dermal fibroblast cells and supported cell attachment and proliferation [99].

Bharathi et al. loaded curcumin in chitosan/PLA nanofibers by using the electrospinning method. The suitability of created nanofibers was studied by antioxidant, drug release and in vitro cytotoxicity studies. In vivo wound healing tests on wounds using a rat model revealed a significant reduction in wound area. The authors concluded that the better healing efficiency could be attributed to the presence of curcumin and chitosan in the fibrous materials [100].

One-pot electrospinning was used to obtain fibrous mats based on PLA and PVP or polyethylene glycol (PEG) loaded with curcumin. The loading of curcumin into the fibers resulted in curcumin shielding from photodestruction and an increase in the mechanical properties of the fibers. In addition, the drug release was facilitated by the formation of hydrogen bonds between curcumin and PVP or PEG. The release of the natural extract provides the antibacterial and anticoagulant activity of the curcumin-loaded mats and prevents adhesion and aggregation of platelets onto the surface of the prepared mats [101].

Electrospun membranes containing curcumin were obtained from poly(L-*co*-D,L-lactic) acid and PVP [102]. It was determined that the dynamic viscosities of the spinning solutions depended on the amount of curcumin, which influenced the average fiber diameter and fiber shape. The authors proved that upon UV-Vis irradiation the amount, physico-chemical and therapeutic properties of curcumin were preserved, revealing the possibility of sterilizing the fibrous biomaterials by UV light.

Pegylated curcumin derivatives were obtained by a direct esterification reaction between poly(ethylene glycol)diacid and curcumin in the presence of *N*,*N*′-dicyclohexylcarbodiimide (PEG600-Curc). The prepared pegylated curcumin derivative is water-soluble. The synthesized product possessed cytotoxic activity against Graffi cell lines along with antibacterial activity [103].

A PHB polymer was used for the design of curcumin-loaded electrospun mats as a wound healing material [104]. The microscopic images of the fibers revealed that the fiber diameter increased with the increase in curcumin concentration. In addition, an increase in the curcumin amount resulted in an increase in the elongation of the fibrous material. The results from the cytotoxicity study show that the samples with a lower curcumin amount showed better biocompatibility. The authors concluded that the electrospun curcumin-loaded PHB mats could be potential candidates for wound healing applications.

In general, the research studies showed the successful incorporation of curcumin in different biopolymers by electrospinning and revealed the perspective for applications of the obtained novel materials as wound dressings as well as for anticancer treatment.

*M. officinalis* L. is a medicinal plant of the *Lamiaceae* family that is known as lemon balm [105]. Many pharmacological studies reveal the diverse inherent properties of *M. officinalis*. Up to now, there have been few studies reporting the incorporation of an *M. officinalis* plant extract into polymer fibers by electrospinning and investigating their characteristic properties. Bioactive nanofibers based on collagen hydrolysate-chitosan and the essential oil of lemon balm (*M. officinalis* L.) and dill (*Anethum graveolens* L.) were obtained by coaxial electrospinning for potential wound dressing applications [106]. The authors report that the synergetic effect of the used essential oils improves the antimicrobial activity of collagen hydrolysate-chitosan nanofibers against the following bacterial, yeast and fungal strains: *S. aureus*, *E. coli*, *Enterococcus faecalis*, *Salmonella typhimurium*, *C. albicans*, *C. glabrata* and *Aspergillus brasiliensis*. The results from the in vivo test showed good biocompatibility of electrospun materials based on collagen hydrolysate/chitosan and containing bioactive compounds (dill and/or lemon balm essential oils). This makes the obtained materials suitable for wound healing applications.

**Figure 6:**
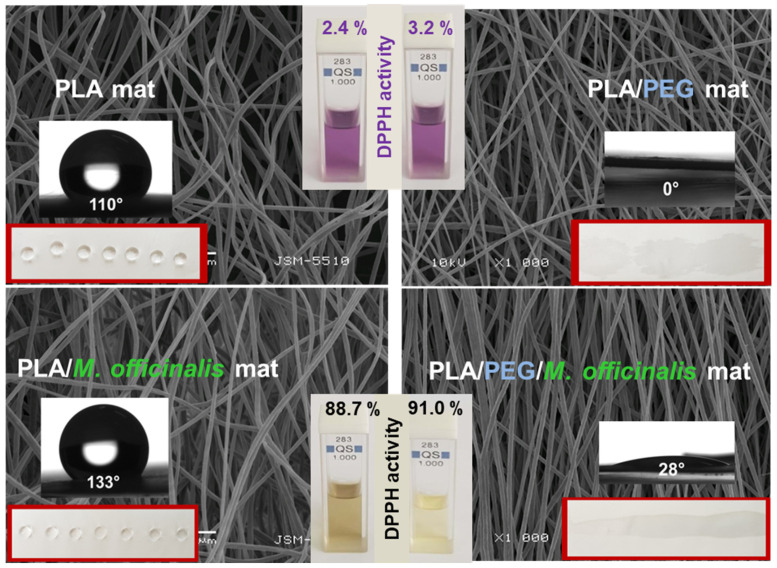
SEM images, water contact angle and antioxidant activity of fibrous mats based on PLA, PLA/PEG, PLA/*M. officinalis* (10 wt%) and PLA/PEG/*M. officinalis* (10 wt%) [107].

Some of us have reported for the first time a study revealing the successful incorporation of *M. officinalis* plant extract into PLA and PLA/PEG fibers by electrospinning [107]. The optimal parameters of the process needed for the fabrication of defect-free hybrid fibers are the following: applied voltage—25 kV, tip to collector distance—15 cm, collector rotation speed—1000 rpm, constant feed rate—3 mL/h, room temperature—21 °C and a relative humidity of 53%. The concentration of the plant extract was varied (0, 5 or 10 wt% in respect to the polymer weight). The measured average fiber diameters of the PLA, PLA/*M. officinalis* (5 wt%) and PLA/*M. officinalis* (10 wt%) were 1370 ± 220 nm, 1398 ± 233 nm and 1506 ± 242 nm, respectively (Figure 6). The slight increase in mean fiber diameter is due to the increase in *M. officinalis* concentration. The presence of the polyether PEG into the hybrid materials resulted in hydrophilization of the resulted electrospun mats. *M. officinalis*-containing fibrous mats possessed high antioxidant activity as determined by the DPPH free radical method. After being in contact with PLA/*M. officinalis* and PLA/PEG/*M. officinalis* fibrous materials, the color of the DPPH solution changed to yellowish and the absorbance of the radical has dropped by 88.7% and 91%, respectively (Figure 6). The reported results revealed the optimal conditions for fabrication of PLA or PLA/PEG fibrous materials incorporated with *M. officinalis* plant extract that were promising candidates for pharmaceutical, cosmetic and biomedical applications.

*Rosmarinus officinalis* (*R. officinalis*) is a plant belonging to the family *Lamiaceae* and native to the Mediterranean region. *R. officinalis* is used as a spice in cooking, as a natural preservative in the food industry and as medicinal plant [108]. This medicinal plant contains diverse bioactive molecules that were responsible for its anti-inflammatory, antioxidant, antimicrobial, antiproliferative, anticancer and protective properties. Several phytocompounds could be isolated from the *R. officinalis* L.: caffeic acid, rosmarinic acid (RA), carnosic acid, chlorogenic acid, monomeric acid, oleanolic acid, ursolic acid, alpha-pinene, camphor, carnosol, eucalyptol, rosmadial, rosmanol, rosmaquinones A and B, secohinokio, and derivatives of eugenol and luteolin [109]. For instance, there is data that, as an excellent antioxidant, RA prevents cell damage and thus lowers the risk of cancer and atherosclerosis. There is no evidence of harm from the use of rosmarinic acid in the literature. However, despite the high therapeutic potential, limited solubility in water and body fluids, chemical instability, poor absorption, rapid metabolism and elimination from the human body determine its low bioavailability, which significantly limits its use in clinical practice as a therapeutic agent [110]. This necessitates the development of new biomaterials as suitable and effective carriers of rosmarinic acid that increase its bioavailability or the finding of new rational solutions and approaches to improve the existing ones. Recently, fibrous polymer materials obtained by electrospinning have been of great interest as carriers of RA [111]. So far, fibrous materials obtained by electrospinning of poly(ε-caprolactone) solutions containing RA and magnetite with the potential application as a drug delivery system have been reported [112]. The electrospinning of CA containing RA in concentrations of 5 and 10% is also reported [113]. A disadvantage of electrospinning in this case is the use of an extremely low solution feeding rate (250 μL/h), which significantly extends the time to obtain the non-woven textile. In addition, some of us have deposited a utility model revealing the creation of a non-woven textile (mat) composition from CA containing RA and a non-ionic polymer. The non-ionogenic water-soluble polymer improves the water solubility of RA and facilitates its release from the nonwoven fabric. The concentration of RA was from 10 to 20 wt% in regard to the weight of the polymers. The created electrospun materials containing RA exhibited high antioxidant activity. The strong antioxidant activity of the obtained fibrous materials from the biopolymer CA, the nonionic water-soluble and the biologically active RA makes them suitable for applications in biomedicine, in the textile industry, in pharmacy, in cell and tissue engineering, in cosmetics for topical application, as well as hygienic, healing and dressing materials, etc. [114].

**Figure 7:**
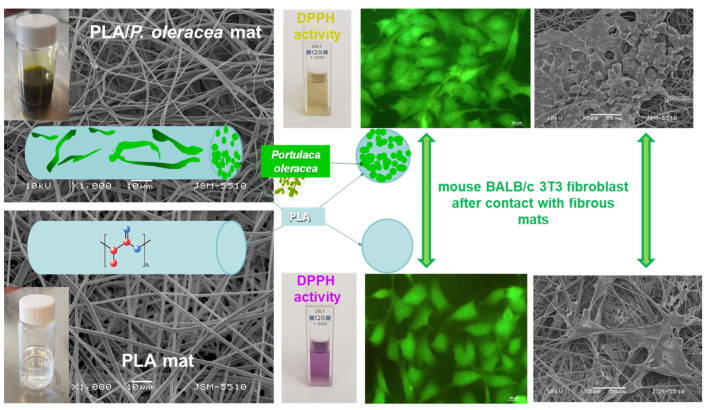
Spinning solution images, SEM micrographs, antioxidant activity, fibroblast staining and cytotoxicity assay of electropun PLA and PLA/*P. oleracea* mats [119].

*Portulaca oleracea* L. (*P. oleracea*) is an annual weed from the family *Portulacaceae* that is native to the Mediterranean region and has spread worldwide. The World Health Organization classified it as one of the most used therapeutic plants [115]. Flavonoids, alkaloids, polysaccharides, fatty acids, terpenoids, sterols, proteins, vitamins and minerals were isolated from this herb [116]. Because of these beneficial and diverse compounds, *P. oleracea* possesses antioxidant, antimicrobial, anti-inflammatory, cardioprotective, neuroprotective, antidiabetic and immunomodulatory activities [117]. Nevertheless, up to now there are only two reports reporting the fabrication of fibrous electrospun materials loaded with *P. oleracea* extract [118,119]. PLA was used to create electrospun materials loaded with *P. oleracea* plant extract obtained by supercritical carbon dioxide. The obtained novel materials were morphologically, physico-chemically, mechanically and biologically characterized. The observation of the obtained fibrous mats by SEM revealed that smooth and defect-free fibers with diameters in the micron scale were obtained (Figure 7). The mechanical properties of the prepared samples were examined, and the results revealed that the tensile strength values of PLA fibrous materials and the hybrid material loaded with *P. oleracea* were very similar. The tensile strength of the PLA/plant extract mat reached ~3.78 MPa while the tensile strength of the PLA fibrous material was ca. 3.9 MPa. Based on these findings we can conclude that the incorporation of the plant extract into the PLA matrix does not result in a decrease in the mechanical properties of the hybrid fibrous material. In addition, the antioxidant activity of the obtained novel electropsun mats was evaluated. It was detected that the PLA/*P. oleracea* exhibited considerable antioxidant activity. Moreover, in vitro studies proved that the electrospun PLA and the PLA/*P. oleracea* materials had no cytotoxic effects. Furthermore, the combination of the biopolymer—PLA with the *P. oleracea* plant extract—promoted cell survival and proliferation of normal mouse fibroblasts. All these results reveal the potential use of the prepared fibrous PLA/*P. oleracea* materials by electrospinning in wound healing applications [118,119].

*Hypericum perforatum (H. perforatum)*, known as St. John’s wort, is a perennial plant with worldwide distribution grown for its medicinal use. There are reports showing the incorporation of *H. perforatum* L. in electrospun dressing materials which are helpful for preventing infections in wounds. A novel double-layer dressing material based on nanofibers was fabricated using poly (L-lactic acid) in the outer layer and a mixture of poly(ethylene) oxide and chitosan loaded with *H. perforatum* [120]. The extract-loaded, fibrous material possessed no cytotoxicity to normal human dermal fibroblast cells along with ability to inhibit *S. aureus* and *P. aeruginosa*. These results reveal the possibility of applying it as an antibacterial wound dressing to treat skin lesions. Furthermore, fibrous mats based on PLA, PVA and chitosan incorporated with the extract were fabricated by emulsion electrospinning of homogeneous gel-like suspensions of different and incompatible polymer solutions [120]. The results revealed the possibility of applying the created material as an antibacterial nanofibrous wound dressing preventing infections and enhancing the wound healing [121].

In addition, the electrospinning method was used to fabricate PVA-based, electrospun wound membranes with hydrolyzed collagen and with different concentrations of *H. perforatum* extract [122]. Due to their composition, ideal porosity and barrier properties, the obtained fibrous dressings could be applied as potential biomedical devices to treat skin injuries in the biomedical field. Moreover, a novel biodegradable polyhydroxybutyrate (PHB) submicron fibrous mat loaded with *Hypericum perforatum* extract using centrifugal spinning technology was fabricated. The results demonstrated strong antibacterial activity of the prepared mats against *E. coli.* The obtained novel hybrid mat possesses the potential to be used in self-cleaning and antibacterial air filters [123].

Up to now, there are some reports revealing the successful incorporation of extracts such as *Chamomile* [124], *Aloe vera* [125,126,127,128], *C. officinalis* [129,130,131], etc. in electrospun fibrous materials. Table 1 presented the plant extracts loaded into polymer materials by electrospinning summarized in Section 5.

Electrospinning is an excellent method for the creation of nanofibrous polymer or hybrid structures, allowing the control of fiber orientation. The fiber properties could be controlled by easily changing the effective parameters of the process. In addition, diverse bioactive compounds (BAC) can be loaded by either mixing with polymers and being electrospun together to form the encapsulated fibers; by electrospraying of BAC/polymer onto the electrospun polymer fibers; or by creating core shell structures by using coaxial electrospinning. In the past decade, the progress in electospinning in laboratory conditions has been significant. However, the industrialization of nanofiber production by electrospinning is developing slowly because the production rate is far lower than the requirement for commercial usage. Therefore, many studies are performed to increase the production speed for diverse systems to be electrospun in commercial scale. Up to now, many companies have been applying commercial electrospinning set-ups to fabricate nanofibrous materials, realizing this huge business opportunity niche. On the other hand, research studies aiming at the fabrication of nanofibrous materials based on plant extracts and polymers are relatively limited and are an emerging field. Plant extracts have a positive effect in electrospinning applications with their biodiversity, ability to sustain biological functionality, and their complex of favorable properties. With the production of nanofibrous structures containing extracts from natural sources, applications in fields such as wound healing, tissue engineering and drug release are increasing every day. This is an extremely promising field that will continue to develop at an exceptionally fast rate in the next few years.

## 6. Conclusions

The present review summarized the achievements in the field of loading different natural plant extracts in nano- and microfibrous polymer materials by the electrospinning method. Most of the presented reports are published in the last 3–4 years. This reveals the nascent and growing interest in this type of hybrid material possessing many desirable chemical and physical properties along with diverse therapeutic effects. Combining the medicinal plant extracts with biopolymers by electrospinning opens the door to the creation of a new generation of complex biomaterials with a variety of applications.

## Figures and Tables

**Table 1:** Plant extracts loaded in electrospun materials.

Plant Extract	Polymer	Reference
*C. longa*	Cellulose acetate/polyvinylpyrrolidone	[92,94]
*C. longa*	Cellulose acetate (CA)	[93]
*C. longa*	Polyvinyl alcohol (PVA)	[95,96,97]
*C. longa*	PVA/CA	[98]
*C. longa*	PLA	[99]
*C. longa*	Chitosan/PLA	[100]
*C. longa*	PLA and PVP or polyethylene glycol (PEG)	[101]
*C. longa*	Poly(L-*co*-D,L-lactic) acid and PVP	[102]
*C. longa*	Poly(ethylene glycol)diacid	[103]
*C. longa*	PHB	[104]
*M. officinalis*	Collagen hydrolysate-chitosan	[106]
*M. officinalis*	PLA and PLA/PEG	[107]
*R. officinalis*	Poly(ε-caprolactone)	[112]
*R. officinalis*	CA	[113]
*R. officinalis*	CA/PEG	[114]
*P. oleracea*	PLA	[118,119]
*H. perforatum*	PLA	[120,121]
*H. perforatum*	PVA	[122,123]
*Chamomile*	Chitosan/PVA and polycaprolactone	[124]
*Aloe vera*	Chitosan/polyethylene oxide	[125]
*Aloe vera*	Poly(ε-caprolactone)	[126]
*Aloe vera*	Chitosan and pullulan	[127]
*Aloe vera*	Chitosan/polyethylene oxide	[128]
*C. officinalis*	Chitosan/PEO	[129]
*C. officinalis*	Polycaprolactone/gelatin	[130]
*C. officinalis*	PVA/Sodium alginate	[131]

## Data Availability

Not applicable.

## Supplementary Materials

The following supporting information can be downloaded at: https://www.mdpi.com/article/10.3390/membranes13100840/s1, Table S1: The benefits of some plant extracts for health. References [132,133,134,135,136,137,138,139,140,141,142,143,144,145,146,147,148,149,150,151,152,153,154,155,156,157,158,159,160,161,162,163,164,165,166,167,168] are cited in the supplementary materials.

## References

[1] Petrovska B. (2012). Historical Review of Medicinal Plants’ Usage. Pharmacogn. Rev..

[2] Teo W.E., Ramakrishna S. (2006). A Review on Electrospinning Design and Nanofibre Assemblies. Nanotechnology.

[3] Wang S., Yap C., He J., Chen C., Wong S., Li X. (2016). Electrospinning: A Facile Technique for Fabricating Functional Nanofibers for Environmental Applications. Nanotechnol. Rev..

[4] Aghababaei F., McClements D.J., Martinez M.M., Hadidi M. (2024). Electrospun Plant Protein-Based Nanofibers in Food Packaging. Food Chem..

[5] Zhao J., Liu Z., Low S.C., Xu Z., Tan S.H. (2023). Electrospinning Technique Meets Solar Energy: Electrospun Nanofiber-Based Evaporation Systems for Solar Steam Generation. Adv. Fiber Mater..

[6] Tipduangta P., Watcharathirawongs W., Waritdecha P., Sirithunyalug B., Leelapornpisid P., Chaiyana W., Goh C.F. (2023). Electrospun Cellulose Acetate/Polyvinylpyrrolidone Fiber Mats as Potential Cosmetic Under-Eye Masks for Caffeine Delivery. J. Drug Deliv. Sci. Technol..

[7] Passaro J., Imparato C., Parida D., Bifulco A., Branda F., Aronne A. (2022). Electrospinning of PVP-Based Ternary Composites Containing SiO_2_ Nanoparticles and Hybrid TiO_2_ Microparticles with Adsorbed Superoxide Radicals. Compos. B Eng..

[8] Zhang M., Xu S., Wang R., Che Y., Han C., Feng W., Wang C., Zhao W. (2023). Electrospun Nanofiber/Hydrogel Composite Materials and Their Tissue Engineering Applications. J. Mater. Sci. Technol..

[9] Wei M., Pan X., Rong L., Dong A., He Y., Song X., Li J. (2020). Polymer Carriers for Controlled Fragrance Release. Mater. Res. Express..

[10] Fox M., Szoka F., Fréchet J. (2009). Soluble Polymer Carriers for the Treatment of Cancer: The Importance of Molecular Architecture. Acc. Chem. Res..

[11] Toncheva A., Spasova M., Paneva D., Manolova N., Rashkov I. (2011). Drug-Loaded Electrospun Polylactide Bundles. J. Bioact. Compat. Polym..

[12] Spasova M., Stoyanova N., Nachev N., Ignatova M., Manolova N., Rashkov I., Georgieva A., Toshkova R., Markova N. (2023). Innovative Fibrous Materials Loaded with 5-Nitro-8-hydroxyquinoline via Electrospinning/Electrospraying Demonstrate Antioxidant, Antimicrobial and Anticancer Activities. Antioxidants.

[13] Atanasov A., Waltenberger B., Pferschy-Wenzig E.-M., Linder T., Wawrosch C., Uhrin P., Temml V., Wang L., Schwaiger S., Heiss E. (2015). Discovery and Resupply of Pharmacologically Active Plant-Derived Natural Products: A Review. Biotechnol. Adv..

[14] Khan T., Ali M., Khan A., Nisar P., Jan S., Afridi S., Shinwari Z. (2019). Anticancer Plants: A Review of the Active Phytochemicals, Applications in Animal Models, and Regulatory Aspects. Biomolecules.

[15] Uttara B., Singh A., Zamboni P., Mahajan R. (2009). Oxidative Stress and Neurodegenerative Diseases: A Review of Upstream and Downstream Antioxidant Therapeutic Options. Curr. Neuropharmacol..

[16] Luengo-Fernandez R., Leal J., Gray A., Sullivan R. (2013). Economic Burden of Cancer Across the European Union: A Population-Based Cost Analysis. Lancet Oncol..

[17] Jha V., Al-Ghamdi S., Li G., Wu M.-S., Stafylas P., Retat L., Card-Gowers J., Barone S., Cabrera C., Garcia Sanchez J. (2023). Global Economic Burden Associated with Chronic Kidney Disease: A Pragmatic Review of Medical Costs for the Inside CKD Research Programme. Adv. Ther..

[18] Srinivasan D., Nathan S., Suresh T., Perumalsamy P. (2001). Antimicrobial Activity of Certain Indian Medicinal Plants used In Folkloric Medicine. J. Ethnopharmacol..

[19] El Maaiden E., Bouzroud S., Nasser B., Moustaid K., El Mouttaqi A., Ibourki M., Boukcim H., Hirich A., Kouisni L., El Kharrassi Y. (2022). A Comparative Study between Conventional and Advanced Extraction Techniques: Pharmaceutical and Cosmetic Properties of Plant Extracts. Molecules.

[20] Marsoul A., Ijjaali M., Oumous I., Bennani B., Boukir A. (2020). Determination of Polyphenol Contents in *Papaver rhoeas* L. Flowers Extracts (Soxhlet, Maceration), Antioxidant and Antibacterial Evaluation. Mater. Today Proc..

[21] Shirsath S., Sable S., Gaikwad S., Sonawane S.H., Saini D., Gogate P. (2017). Intensification of Extraction of Curcumin from *Curcuma amada* Using Ultrasound Assisted Approach: Effect of Different Operating Parameters. Ultrason. Sonochem..

[22] Bachtler S., Bart H.-J. (2021). Increase the Yield of Bioactive Compounds from Elder Bark and Annatto Seeds Using Ultrasound and Microwave Assisted Extraction Technologies. Food. Bioprod. Process..

[23] Bajpai V., Majumder R., Park J. (2016). Isolation and Purification of Plant Secondary Metabolites Using Column-Chromatographic Technique. Bangladesh J. Pharmacol..

[24] Ali K., Ali A., Khan M.N., Rahman S., Faizi S., Ali M., Khalifa S., El-Seedi H., Musharraf S. (2021). Rapid Identification of Common Secondary Metabolites of Medicinal Herbs Using High-Performance Liquid Chromatography with Evaporative Light Scattering Detector in Extracts. Metabolites.

[25] Hassaan Y., Handoussa H., El-Khatib A., Linscheid M., El Sayed N. (2014). Evaluation of Plant Phenolic Metabolites as a Source of Alzheimer’s Drug Leads. BioMed Res. Int..

[26] Rahmati B., Kiasalari Z., Roghani M., Khalili M., Ansari F. (2017). Antidepressant and Anxiolytic Activity of *Lavandula officinalis* Aerial Parts Hydroalcoholic Extract in Scopolamine-Treated Rats. Pharm. Biol..

[27] Saleem U., Hussain L., Shahid F., Anwar F., Chauhdary Z., Zafar A. (2022). Pharmacological Potential of the Standardized Methanolic Extract of *Prunus armeniaca* L. in the Haloperidol-Induced Parkinsonism Rat Model. Evid. Based Complement. Alternat. Med..

[28] Cao Y.-L., Lin J.-H., Hammes H.-P., Zhang C. (2022). Flavonoids in Treatment of Chronic Kidney Disease. Molecules.

[29] Khan M., Kassianos A., Hoy W., Alam K., Healy H., Gobe G. (2022). Promoting Plant-Based Therapies for Chronic Kidney Disease. J. Evid. Based Integr. Med..

[30] Liwa A., Jaka H. (2016). Renal Diseases and Use of Medicinal Herbal Extracts: A Concise Update of Reported Literature in Africa. J. Nephrol. Renal Ther..

[31] Jahanban-Esfahlan A., Modaeinama S., Abasi M., Abbasi M., Jahanban-Esfahlan R. (2015). Anti Proliferative Properties of Melissa officinalis in Different Human Cancer Cells. Asian Pac. J. Cancer Prev..

[32] Gull N., Arshad F., Naikoo G., Hassan I., Pedram M., Ahmad A., Aljabali A., Mishra V., Satija S., Charbe N. (2022). Recent Advances in Anticancer Activity of Novel Plant Extracts and Compounds from *Curcuma longa* in Hepatocellular Carcinoma. J. Gastrointest. Cancer.

[33] Gomes de Souza P., Rosenthal A., Ayres E., Teodoro A. (2022). Potential Functional Food Products and Molecular Mechanisms of *Portulaca oleracea* L. on Anticancer Activity: A Review. Oxid. Med. Cell. Longev..

[34] Roozbeh N., Darvish L., Abdi F. (2017). Hypoglycemic Effects of *Acacia nilotica* in Type Ii Diabetes: A Research Proposal. BMC Res. Notes.

[35] Mukundi M.J., Piero N.M., Mwaniki N.E.N., Murugi N.J., Daniel A.S., Peter G.K., Alice M.N. (2015). Antidiabetic Effects of Aqueous Leaf Extracts of *Acacia niloticain* Alloxan Induced Diabetic Mice. J. Diabetes Metab..

[36] Dulić M., Ciganović P., Vujić L., Zovko Končić M. (2019). Antidiabetic and Cosmeceutical Potential of Common Barbery (*Berberis vulgaris* L.) Root Bark Extracts Obtained by Optimization of ‘Green’ Ultrasound-Assisted Extraction. Molecules.

[37] Aziz T., Hussain S., Mahwi T., Ahmed Z., Rahman H., Rasedee A. (2018). The Efficacy and Safety of Ginkgo Biloba Extract as an Adjuvant in Type 2 Diabetes Mellitus Patients Ineffectively Managed with Metformin: A Double-Blind, Randomized, Placebo-Controlled Trial. Drug Des. Devel. Ther..

[38] Petrisor G., Motelica L., Craciun L., Oprea O., Ficai D., Ficai A. (2022). Melissa officinalis: Composition, Pharmacological Effects and Derived Release Systems—A Review. Int. J. Mol. Sci..

[39] Asadi A., Shidfar F., Safari M., Hosseini A., Huseini H., Heidari I., Rajab A. (2019). Efficacy of *Melissa officinalis* L. (Lemon Balm) Extract on Glycemic Control and Cardiovascular Risk Factors in Individuals with Type 2 Diabetes: A Randomized, Double-Blind, Clinical Trial. Phytother. Res..

[40] Li Y., Ding Y., Xiao W., Zhu J.-B. (2021). Investigation on the Active Ingredient and Mechanism of *Cannabis sativa* L. For Treating Epilepsy Based on Network Pharmacology. Biotechnol. Biotechnol. Equip..

[41] Jalali J., Rahbardar M. (2023). Ameliorative Effects of *Portulaca oleracea* L. (Purslane) and Its Active Constituents on Nervous System Disorders: A Review. Iran. J. Basic. Med. Sci..

[42] Kapoor L. (2017). Handbook of Ayurvedic Medicinal Plants: Herbal Reference Library.

[43] Boskabady M., Shakeri F., Naghdi F. (2020). Studies in Natural Products Chemistry Chapter 7—The Effects of *Curcuma longa* L. Its Constituents in Respiratory Disorders and Molecular Mechanisms of Their Action. Studies in Natural Products Chemistry.

[44] Londhe V., Gavasane A., Nipate S., Bandawane D., Chaudhari P. (2011). Role of Garlic (*Allium sativum*) In Various Diseases: An Overview. Angiogenesis.

[45] Sanna C., Marengo A., Acquadro S., Caredda A., Lai R., Corona A., Tramontano E., Rubiolo P., Esposito F. (2021). In Vitro Anti-HIV-1 Reverse Transcriptase and Integrase Properties of *Punica granatum* L. Leaves, Bark, and Peel Extracts and Their Main Compounds. Plants.

[46] Mahdavi M.A., Seyedsadjadi N., Javadivala Z. (2021). Potential Effects of Pomegranate (*Punica granatum*) On Rheumatoid Arthritis: A Systematic Review. Int. J. Clin. Pract..

[47] Palshetkar A., Pathare N., Jadhav N., Pawar M., Wadhwani A., Kulkarni S., Singh K. (2020). In Vitro Anti-HIV Activity of Some Indian Medicinal Plant Extracts. BMC Complement. Med. Ther..

[48] Kumar R., Nair V., Singh S., Gupta Y. (2015). In Vivo Antiarthritic Activity of *Rosa centifolia* L. Flower Extract. Ayu.

[49] Baranwal J., Barse B., Fais A., Delogu G.L., Kumar A. (2022). Biopolymer: A Sustainable Material for Food and Medical Applications. Polymers.

[50] Mohan O., Oluwafemi S., Kalarikkal N., Thomas S., Songca S.P. (2016). Biopolymers—Application in Nanoscience and Nanotechnology. Recent Advances in Biopolymers.

[51] Lisitsyn A., Semenova A., Nasonova V., Polishchuk E., Revutskaya N., Kozyrev I., Kotenkova E. (2021). Approaches in Animal Proteins and Natural Polysaccharides Application for Food Packaging: Edible Film Production and Quality Estimation. Polymers.

[52] Fatima A., Yasir S., Khan M., Manan S., Ullah M., Ul-Islam M. (2021). Plant Extract-Loaded Bacterial Cellulose Composite Membrane for Potential Biomedical Applications. J. Bioresour. Bioprod..

[53] Rajeswari A., Christy J., Swathi E., Pius A. (2020). Fabrication of Improved Cellulose Acetate-Based Biodegradable Films for Food Packaging Applications. Environ. Toxicol. Chem..

[54] Hassanloofard Z., Gharekhani M., Zandi M., Ganjloo A., Roufegarinejad L. (2023). Fabrication and Characterization of Cellulose Acetate Film Containing *Falcaria vulgaris* Extracta. Cellulose.

[55] Gradinaru L., Barbalata-Mandru M., Enache A., Rimbu C., Badea G., Aflori M. (2023). Chitosan Membranes Containing Plant Extracts: Preparation, Characterization and Antimicrobial Properties. Int. J. Mol. Sci..

[56] Benković M., Sarić I., Jurinjak Tušek A., Jurina T., Gajdoš Kljusurić J., Valinger D. (2021). Analysis of the Adsorption and Release Processes of Bioactives from Lamiaceae Plant Extracts on Alginate Microbeads. Food Bioprocess. Technol..

[57] Kumari A., Kumar V., Yadav S.K. (2012). Plant Extract Synthesized PLA Nanoparticles for Controlled and Sustained Release of Quercetin: A Green Approach. PLoS ONE.

[58] Lahmar A., Rjab M., Sioud F., Selmi M., Salek A., Kilani-Jaziri S., Ghedira L.C. (2022). Design of 3D Hybrid Plant Extract/Marine and Bovine Collagen Matrixes as Potential Dermal Scaffolds for Skin Wound Healing. Sci. World J..

[59] Gavan A., Colobatiu L., Hanganu D., Bogdan C., Olah N.K., Achim M., Mirel S. (2022). Development and Evaluation of Hydrogel Wound Dressings Loaded with Herbal Extracts. Processes.

[60] Wang W.-C., Cheng Y.-T., Estroff B. (2021). Electrostatic Self-Assembly of Composite Nanofiber Yarn. Polymers.

[61] Xie Y., Kocaefe D., Chen C., Kocaefe Y. (2016). Review of Research on Template Methods in Preparation of Nanomaterials. J. Nanomater..

[62] Wei J., Liang W., Zhang J. (2023). Preparation of Mechanically Stable Superamphiphobic Coatings via Combining Phase Separation of Adhesive and Fluorinated SiO_2_ for Anti-Icing. Nanomaterials.

[63] Yadavalli N., Asheghali D., Tokarev A., Zhang W., Xie J., Minko S. (2020). Gravity Drawing of Micro- and Nanofibers for Additive Manufacturing of Well-Organized 3D-Nanostructured Scaffolds. Small.

[64] Botta L., Teresi R., Titone V., Salvaggio G., La Mantia F., Lopresti F. (2021). Use of Biochar as Filler for Biocomposite Blown Films: Structure-Processing-Properties Relationships. Polymers.

[65] Huang Z.-M., Zhang Y.-Z., Kotaki M., Ramakrishna S. (2003). A Review on Polymer Nanofibers by Electrospinning and Their Applications in Nanocomposites. Compos. Sci. Technol..

[66] Islam S., Ang B., Andriyana A., Afifi A. (2019). A Review on Fabrication of Nanofibers via Electrospinning and Their Applications. SN Appl. Sci..

[67] Reneker D., Yarin A. (2008). Electrospinning Jets and Polymer Nanofibers. Polymer.

[68] Al-Abduljabbar A., Farooq I. (2023). Electrospun Polymer Nanofibers: Processing, Properties, and Applications. Polymers.

[69] Bhushani J., Anandharamakrishnan C. (2014). Electrospinning and Electrospraying Techniques: Potential Food Based Applications. Trends Food Sci. Technol..

[70] Fokin N., Grothe T., Mamun A., Trabelsi M., Klöcker M., Sabantina L., Döpke C., Blachowicz T., Hütten A., Ehrmann A. (2020). Magnetic Properties of Electrospun Magnetic Nanofiber Mats after Stabilization and Carbonization. Materials.

[71] Ignatova M., Rashkov I., Manolova N. (2013). Drug-Loaded Electrospun Materials in Wound-Dressing Applications and in Local Cancer Treatment. Expert. Opin. Drug. Deliv..

[72] Ignatova M., Nachev N., Spasova M., Manolova N., Rashkov I., Naydenov M. (2022). Electrospun 5-Chloro-7-iodo-8-hydroxyquinoline (Clioquinol)-Containing Poly(3-hydroxybutyrate)/Polyvinylpyrrolidone Antifungal Materials Prospective as Active Dressings against Esca. Polymers.

[73] Korina E., Stoilova O., Manolova N., Rashkov I. (2018). Polymer Fibers with Magnetic Core Decorated with Titanium Dioxide Prospective for Photocatalytic Water Treatment. J. Environ. Chem. Eng..

[74] Shenoy S., Bates W., Frisch H., Wnek G. (2005). Role of Chain Entanglements on Fiber Formation During Electrospinning of Polymer Solutions: Good Solvent, Non-specific Polymer–Polymer Interaction Limit. Polymer.

[75] Spasova M., Manolova N., Paneva D., Rashkov I. (2004). Preparation of Chitosan Containing Nanofibers by Electrospinning Chitosan/Poly(Ethylene Oxide) Mixed Solutions. e-Polymers.

[76] Stoyanova N., Paneva D., Mincheva R., Toncheva A., Manolova N., Dubois P., Rashkov I. (2014). Poly(L-Lactide) And Poly(Butylene Succinate) Immiscible Blends: From Electrospinning to Biologically Active Materials. Mater. Sci. Eng. C.

[77] Tarus B., Fadel N., Al-Oufy A., El-Messiry M. (2016). Effect of Polymer Concentration on the Morphology and Mechanical Characteristics of Electrospun Cellulose Acetate and Poly (Vinyl Chloride) Nanofiber Mats. Alex. Eng. J..

[78] Bhardwaj N., Kundu S. (2010). Electrospinning: A Fascinating Fiber Fabrication Technique. Biotechnol. Adv..

[79] Topuz F., Abdulhamid M., Holtzl T., Szekely G. (2021). Nanofiber Engineering of Microporous Polyimides Through Electrospinning: Influence of Electrospinning Parameters and Salt Addition. Mater. Des..

[80] Haider A., Haider S., Kang I.-K. (2018). A Comprehensive Review Summarizing the Effect of Electrospinning Parameters and Potential Applications of Nanofibers in Biomedical and Biotechnology. Arab. J. Chem..

[81] Laudenslager M., Sigmund W. (2012). Electrospinning. Encyclopedia of Nanotechnology.

[82] Angammana C., Jayaram S. (2015). Fundamentals of Electrospinning and Processing Technologies. Part. Sci. Technol..

[83] Xue J., Wu T., Dai Y., Xia Y. (2019). Electrospinning and Electrospun Nanofibers: Methods, Materials, and Applications. Chem. Rev..

[84] Megelski S., Stephens J., Chase D., Rabolt J. (2002). Micro- And Nanostructured Surface Morphology on Electrospun Polymer Fibers. Macromolecules.

[85] Lee J., Choi K., Ghim H., Kim S., Chun D., Kim H., Lyoo W. (2004). Role of Molecular Weight of Atactic Poly(Vinyl Alcohol) (PVA) In the Structure and Properties of PVA Nanofabric Prepared by Electrospinning. J. Appl. Polym. Sci..

[86] Jabur A., Abbas L., Aldain S. (2017). Effects of Ambient Temperature and Needle to Collector Distance on PVA Nanofibers Diameter Obtained From Electrospinning Technique. Eng. Technol. J..

[87] Yang G.-Z., Li H.-P., Yang J.-H., Wan J., Yu D.-G. (2017). Influence of Working Temperature on The Formation of Electrospun Polymer Nanofibers. Nanoscale Res. Lett..

[88] Park B.K., Um I.C. (2021). Effect of Relative Humidity on the Electrospinning Performance of Regenerated Silk Solution. Polymers.

[89] Zander N. (2013). Hierarchically Structured Electrospun Fibers. Polymers.

[90] Nayak R., Padhye R. (2017). Nano Fibres by Electro Spinning, Properties and Applications. J. Text. Eng. Fash. Technol..

[91] Subrahmanya T., Arshad A., Lin P., Widakdo J., Makari H., Austria H., Hu C.-C., Lai J.-Y., Hung W.-S. (2021). A Review of Recent Progress in Polymeric Electrospun Nanofiber Membranes in Addressing Safe Water Global Issues. RSC Adv..

[92] Tsekova P., Spasova M., Manolova N., Markova N., Rashkov I. (2017). Electrospun Curcumin-Loaded Cellulose Acetate/Polyvinylpyrrolidone Fibrous Materials with Complex Architecture and Antibacterial Activity. Mater. Sci. Eng. C.

[93] Suwantong O., Opanasopit P., Ruktanonchai U., Supaphol P. (2007). Electrospun Cellulose Acetate Fiber Mats Containing Curcumin and Release Characteristic of the Herbal Substance. Polymer.

[94] Tsekova P., Spasova M., Manolova N., Rashkov I., Markova N., Georgieva A., Toshkova R. (2018). Electrospun Cellulose Acetate Membranes Decorated with Curcumin-PVP Particles: Preparation, Antibacterial and Antitumor Activities. J. Mater. Sci. Mater. Med..

[95] Sun X.-Z., Williams G., Hou X.-X., Zhu L.-M. (2013). Electrospun Curcumin-Loaded Fibers with Potential Biomedical Applications. Carbohydr. Polym..

[96] Mahmud M., Zaman S., Perveen A., Jahan R., Islam F., Arafat M. (2020). Controlled Release of Curcumin from Electrospun Fiber Mats with Antibacterial Activity. J. Drug Deliv. Sci. Technol..

[97] Shahid A., Ali A., Uddin N., Miah S., Islam S., Mohebbullah M., Jamal M. (2021). Antibacterial Wound Dressing Electrospun Nanofibrous Material from Polyvinyl Alcohol, Honey and Curcumin longa Extract. J. Ind. Text..

[98] Gaydhane M., Kanuganti J., Sharma C. (2020). Honey and Curcumin Loaded Multilayered Polyvinylalcohol/Cellulose Acetate Electrospun Nanofibrous Mat for Wound Healing. J. Mater. Res..

[99] Pankongadisak P., Sangklin S., Chuysinuan P., Suwantong O., Supaphol P. (2019). The Use of Electrospun Curcumin-Loaded Poly(L-Lactic Acid) Fiber Mats as Wound Dressing Materials. Drug Deliv. Sci. Technol..

[100] Dhurai B., Saraswathy N., Maheswaran R., Sethupathi P., Vanitha P., Vigneshwaran S., Rameshbabu V. (2013). Electrospinning of Curcumin Loaded Chitosan/Poly (Lactic Acid) Nanofilm and Evaluation of Its Medicinal Characteristics. Front. Mater. Sci..

[101] Yakub G., Toncheva A., Manolova N., Rashkov I., Danchev D., Kussovski V. (2016). Electrospun Polylactide-Based Materials for Curcumin Release: Photostability, Antimicrobial Activity, and Anticoagulant Effect. J. Appl. Polym. Sci..

[102] Yakub G., Toncheva A., Kussovski V., Toshkova R., Georgieva A., Nikolova E., Manolova N., Rashkov I. (2020). Curcumin-PVP Loaded Electrospun Membranes with Conferred Antibacterial and Antitumoral Activities. Fibers Polym..

[103] Yakub G., Manolova N., Rashkov I., Markova N., Toshkova R., Georgieva A., Mincheva R., Toncheva A., Raquez J.-M., Dubois P. (2022). Pegylated Curcumin Derivative: Water-Soluble Conjugates with Antitumor and Antibacterial Activity. ACS Omega.

[104] Ghavami R., Biazar E., Taleghani A., Keshel S. (2020). Design of Curcumin-Loaded Electrospun Polyhydroxybutyrate Mat as a Wound Healing Material. Nano Biomed. Eng..

[105] Papoti V.T., Totomis N., Atmatzidou A., Zinoviadou K., Androulaki A., Petridis D., Ritzoulis C. (2019). Phytochemical Content of *Melissa officinalis* L. Herbal Preparations Appropriate for Consumption. Processes.

[106] Râpa M., Gaidau C., Mititelu-Tartau L., Berechet M.-D., Berbecaru A.C., Rosca I., Chiriac A.P., Matei E., Predescu A.-M., Predescu C. (2021). Bioactive Collagen Hydrolysate-Chitosan/Essential Oil Electrospun Nanofibers Designed for Medical Wound Dressings. Pharmaceutics.

[107] Stoyanova N., Spasova M., Manolova N., Rashkov I., Kamenova-Nacheva M., Staleva P., Tavlinova-Kirilova M. (2023). Electrospun PLA-Based Biomaterials Loaded with Melissa officinalis Extract with Strong Antioxidant Activity. Polymers.

[108] de Oliveira J., Camargo S.E., de Oliveira L. (2019). *Rosmarinus officinalis* L. (Rosemary) As Therapeutic and Prophylactic Agent. J. Biomed. Sci..

[109] Borges R., Ortiz B.L., Pereira A.C., Keita H., Carvalho J.C. (2019). Rosmarinus officinalis Essential Oil: A Review of Its Phytochemistry, Anti-inflammatory Activity, and Mechanisms of Action Involved. J. Ethnopharmacol..

[110] Di Lorenzo C., Colombo F., Biella S., Stockley C., Restani P. (2021). Polyphenols and Human Health: The Role of Bioavailability. Nutrients.

[111] Wen P., Zong M.-H., Linhardt R.J., Feng K., Wu H. (2017). Electrospinning: A Novel Nano-Encapsulation Approach for Bioactive Compounds. Trends Food Sci. Technol..

[112] Saad E., El Gohary N., El-Shenawy B., Handoussa H., Klingner A., Elwi M., Hamed Y., Khalil I., El Nashar R., Mizaikoff B. (2020). Fabrication of Magnetic Molecularly Imprinted Beaded Fibers for Rosmarinic Acid. Nanomaterials.

[113] Vatankhah E. (2018). Rosmarinic Acid-Loaded Electrospun Nanofibers: In Vitro Release Kinetic Study and Bioactivity Assessment. Eng. Life Sci..

[114] Spasova-Todorova M., Stoyanova N., Stoilova O. Composition of non-woven fabric (mat) containing rosmarinic acid. No 5817/10/10/2023.

[115] Azuka O., Mary A., Abu O. (2014). A Review on *Portulaca oleracea* (Purslane) Plant- Its Nature and Biomedical Benefits. Int. J. Biomed. Res..

[116] Zhou Y.-X., Xin H.-L., Rahman K., Wang S.-J., Peng C., Zhang H. (2015). *Portulaca oleracea* L.: A Review of Phytochemistry and Pharmacological Effects. BioMed Res. Int..

[117] Iranshahy M., Javadi B., Iranshahi M., Jahanbakhsh S., Mahyari S., Hassani F., Karimi G. (2017). Review of Traditional Uses, Phytochemistry and Pharmacology of *Portulaca oleracea* L.. J. Ethnopharmacol..

[118] Spasova M., Stoyanova N., Manolova N., Rashkov I., Taneva S., Momchilova S., Georgieva A. (2022). Facile Preparation of Novel Antioxidant Fibrous Material Based on Natural Plant Extract from *Portulaca oleracea* and PLA by Electrospinning for Biomedical Applications. Polym. Int..

[119] Stoyanova N., Spasova M., Manolova N., Rashkov I., Taneva S., Momchilova S., Georgieva A. (2023). Physico-Chemical, Mechanical, and Biological Properties of Polylactide/Portulaca oleracea Extract Electrospun Fibers. Membranes.

[120] Mouro C., Gomes A.P., Gouveia I.C. (2020). Double-Layer PLLA/PEO_chitosan Nanofibrous Mats Containing *Hypericum perforatum* L. As an Effective Approach for Wound Treatment. Polym. Adv. Technol..

[121] Mouro C., Gomes A.P., Gouveia I.C. (2023). Emulsion Electrospinning of PLLA/PVA/Chitosan with *Hypericum perforatum* L. as an Antibacterial Nanofibrous Wound Dressing. Gels.

[122] García-Hernández A., Morales-Sánchez E., Berdeja-Martínez B., Escamilla-García M., Salgado-Cruz M.P., Rentería-Ortega M., Farrera-Rebollo R.R., Vega-Cuellar M., Calderón-Domínguez G. (2022). PVA-Based Electrospun Biomembranes with Hydrolyzed Collagen and Ethanolic Extract of *Hypericum perforatum* for Potential Use as Wound Dressing: Fabrication and Characterization. Polymers.

[123] Beran M., Horna A., Vorisek V., Berkova E., Korinkova R., Trousil V., Hrubanova M. (2022). Antimicrobial Polyhydroxybutyrate Submicron Fiber Mat Loaded with Extract of *Hypericum perforatum*. J. Plant Biotechnol..

[124] Shokrollahi M., Hajir Bahrami S., Haghbin Nazarpak M., Solouk A. (2020). Multilayer Nanofibrous Patch Comprising Chamomile Loaded Carboxyethyl Chitosan/Poly(Vinyl Alcohol) And Polycaprolactone as a Potential Wound Dressing. Int. J. Biol. Macromol..

[125] Nikbakht M., Salehi M., Rezayat S.M., Majidi R.F. (2020). Various Parameters in the Preparation of Chitosan/Polyethylene Oxide Electrospun Nanofibers Containing Aloe Vera Extract for Medical Applications. Nanomed. J..

[126] Baghersad S., Bahrami H., Mohammadi M., Mojtahedi M., Milan P. (2018). Development of Biodegradable Electrospun Gelatin/Aloe-Vera/Poly(ε-Caprolactone) Hybrid Nanofibrous Scaffold for Application as Skin Substitutes. Mater. Sci. Eng. C.

[127] Barbosa R., Villarreal A., Rodriguez C., De Leon H., Gilkerson R., Lozano K. (2021). Aloe Vera Extract-Based Composite Nanofibers for Wound Dressing Applications. Mater. Sci. Eng. C.

[128] Pathalamuthu P., Siddharthan A., Giridev V.R., Victoria V., Thangam R., Sivasubramanian S., Savariar V., Hemamalini T. (2019). Enhanced Performance of Aloe Vera Incorporated Chitosan-Polyethylene Oxide Electrospun Wound Scaffold Produced Using Novel Spirograph Based Collector Assembly. Int. J. Biol. Macromol..

[129] Kharat Z., Goushki M.A., Sarvian N., Asad S., Dehghan M.M., Kabiri M. (2021). Chitosan/PEO Nanofibers Containing *Calendula officinalis* Extract: Preparation, Characterization, In Vitro and In Vivo Evaluation for Wound Healing Applications. Int. J. Pharm..

[130] Azizi M., Azimzadeh M., Afzali M., Alafzadeh M., Mirhosseini S.H. (2018). Characterization and Optimization of Using *Calendula officinalis* Extract in Fabrication of Polycaprolactone-Gelatin Electrospun Nanofibers for Wound Dressing Applications. J. Adv. Mater. Process..

[131] Tahami S., Nemati N., Keshvari H., Khorasani M. (2020). Effect of Electrical Potential on the Morphology of Polyvinyl Alcohol/Sodium Alginate Electrospun Nanofibers, Containing Herbal Extracts of Calendula officinalis for Using in Biomedical Applications. J. Mod. Process. Manuf. Prod..

[132] Hidalgo-Báez D., Ricardi M., Gaviria J., Estrada J. (1999). Aportes a la Etnofarmacología de Los Páramos Venezolanos. Ciencia.

[133] Alerico G.C., Beckenkamp A., Vignoli-Silva M., Buffon A., von Poser G.L. (2015). Proliferative Effect of Plants Used for Wound Healing in Rio Grande Do Sul State, Brazil. J. Ethnopharmacol..

[134] Espiña D.C., Carvalho F.B., Zanini D., Schlemmer J.B., Coracini J.D., Rubin M.A., Morsch V.M., Schetinger M.R.C., Leal D.B.R., Baiotto C.R. (2012). A More Accurate Profile of *Achyrocline satureioides* Hypocholesterolemic Activity. Cell Biochem. Funct..

[135] Obulesu M., Rao D.M. (2011). Effect of Plant Extracts on Alzheimer’s Disease: An Insight into Therapeutic Avenues. J. Neurosci. Rural. Pract..

[136] Chiari M.E., Joray M.B., Ruiz G., Palacios S.M., Carpinella M.C. (2010). Tyrosinase Inhibitory Activity of Native Plants from Central Argentina: Isolation of an Active Principle from Lithrea Molleoides. Food Chem..

[137] Carpinella M.C., Andrione D.G., Ruiz G., Palacios S.M. (2009). Screening for Acetylcholinesterase Inhibitory Activity in Plant Extracts from Argentina. Phytother. Res..

[138] Lamichhane G., Pandey J., Devkota H.P. (2023). Bioactive Chemical Constituents and Pharmacological Activities of *Ponciri fructus*. Molecules.

[139] Kuete V., Metuno R., Ngameni B., Tsafack A.M., Ngandeu F., Fotso G.W., Bezabih M., Etoa F.X., Ngadjui B.T., Abegaz B.M. (2007). Antimicrobial Activity of the Methanolic Extracts and Compounds from *Treculia obovoidea* (Moraceae). J. Ethnopharmacol..

[140] Raafat B.M., Alsanie W.F., Thobaity A.A., Alamri A.S., Elesawy B.H., Dahlawi H. (2021). A Combined Protective Dose of *Angelica archangelica* and *Ginkgo biloba* Restores Normal Functional Hemoglobin Derivative Levels in Rabbits after Oxidative Stress Induced by Gallium-68. Appl. Sci..

[141] Fraternale D., Teodori L., Rudov A., Prattichizzo F., Olivieri F., Guidarelli A., Albertini M.C. (2018). The in Vitro Activity of *Angelica archangelica* L. Essential Oil on Inflammation. J. Med. Food.

[142] Drever B.D., Anderson W.G., Riedel G., Kim D.H., Ryu J.H., Choi D.Y., Platt B. (2008). The Seed Extract of *Cassia obtusifolia* Offers Neuroprotection to Mouse Hippocampal Cultures. J. Pharmacol. Sci..

[143] Ali M.Y., Jannat S., Jung H.A., Min B.-S., Paudel P., Choi J.S. (2018). Hepatoprotective Effect of *Cassia obtusifolia* Seed Extract and Constituents Against Oxidative Damage Induced by Tert -Butyl Hydroperoxide in Human Hepatic HEPG2 Cells. J. Food Biochem..

[144] Kitanaka S., Takido M. (1986). Studies on the Constituents in the Roots of *Cassia obtusifolia* L. and the Antimicrobial Activities of Constituents of the Roots and the Seeds. Yakugaku Zasshi.

[145] Rastogi S., Pandey M.M., Rawat A.K. (2011). An Ethnomedicinal, Phytochemical and Pharmacological Profile of *Desmodium gangeticum* (L.) DC. and *Desmodium adscendens* (Sw.) DC. J. Ethnopharmacol..

[146] Bora M., Mandal S., Singh P.K., Das H., Bora G.K., Bora D., Baruah D., Gautam M.K. (2024). Phytochemical and Pharmacological Profile of *Desmodium gangeticum* (L.) DC.: A Comprehensive Review. Curr. Tradit. Med..

[147] Hamidpour M., Hamidpour R., Hamidpour S., Shahlari M. (2014). Chemistry, Pharmacology, and Medicinal Property of Sage (*Salvia*) to Prevent and Cure Illnesses such as Obesity, Diabetes, Depression, Dementia, Lupus, Autism, Heart Disease, and Cancer. J. Tradit. Complement. Med..

[148] Ezema C.A., Ezeorba T.P.C., Aguchem R.N., Okagu I.U. (2022). Therapeutic Benefits of *Salvia* Species: A Focus on Cancer and Viral Infection. Heliyon.

[149] Leone A., Spada A., Battezzati A., Schiraldi A., Aristil J., Bertoli S. (2015). Cultivation, Genetic, Ethnopharmacology, Phytochemistry and Pharmacology of *Moringa oleifera* Leaves: An Overview. Int. J. Mol. Sci..

[150] Leone A., Spada A., Battezzati A., Schiraldi A., Aristil J., Bertoli S. (2016). *Moringa oleifera* Seeds and Oil: Characteristics and Uses for Human Health. Int. J. Mol. Sci..

[151] Ventura A.C.S.S.B., de Paula T., Gonçalves J.P., da Silva Soley B., Cretella A.B.M., Otuki M.F., Cabrini D.A. (2021). The Oil from *Moringa oleifera* Seeds Accelerates Chronic Skin Wound Healing. Phytomed. Plus.

[152] Mahadevan S., Park Y. (2008). Multifaceted Therapeutic Benefits of *Ginkgo biloba* L.: Chemistry, Efficacy, Safety, and Uses. J. Food Sci..

[153] Prusinowska R., Śmigielski K. (2014). Composition, Biological Properties and Therapeutic Effects of Lavender (*Lavandula angustifolia* L). A Review. Herba Pol..

[154] Kitic D., Miladinovic B., Randjelovic M., Szopa A., Sharifi-Rad J., Calina D., Seidel V. (2022). Anticancer Potential and Other Pharmacological Properties of *Prunus armeniaca* L.: An Updated Overview. Plants.

[155] Wang L., Zhang R.-M., Liu G.-Y., Wei B.-L., Wang Y., Cai H.-Y., Li F.-S., Xu Y.-L., Zheng S.-P., Wang G. (2010). Chinese Herbs in Treatment of Influenza: A Randomized, Double-Blind, Placebo-Controlled Trial. Respir. Med..

[156] Auyeung K.K., Han Q.-B., Ko J.K. (2016). *Astragalus membranaceus*: A Review of its Protection Against Inflammation and Gastrointestinal Cancers. Am. J. Chin. Med..

[157] Panda A.K., Swain K.C. (2011). Traditional Uses and Medicinal Potential of *Cordyceps sinensis* of Sikkim. J. Ayurveda Integr. Med..

[158] Luan F., Wu Q., Yang Y., Lv H., Liu D., Gan Z., Zeng N. (2020). Traditional Uses, Chemical Constituents, Biological Properties, Clinical Settings, and Toxicities of *Abelmoschus manihot* L.: A Comprehensive Review. Front. Pharmacol..

[159] Nassiri-Asl M., Hosseinzadeh H. (2016). Review of the Pharmacological Effects of *Vitis vinifera* (Grape) and its Bioactive Constituents: An Update. Phytother. Res..

[160] Mao Q.-Q., Xu X.-Y., Cao S.-Y., Gan R.-Y., Corke H., Beta T., Li H.-B. (2019). Bioactive Compounds and Bioactivities of Ginger (*Zingiber officinale* Roscoe). Foods.

[161] Sonfack C.S., Nguelefack-Mbuyo E.P., Kojom J.J., Lappa E.L., Peyembouo F.P., Fofié C.K., Nolé T., Nguelefack T.B., Dongmo A.B. (2021). The Aqueous Extract from the Stem Bark of *Garcinia lucida* Vesque (Clusiaceae) Exhibits Cardioprotective and Nephroprotective Effects in Adenine-Induced Chronic Kidney Disease in Rats. Evid. Based Complement. Alternat. Med..

[162] Srivastava R., Srivastava V., Singh A. (2023). Multipurpose Benefits of an Underexplored Species Purslane (*Portulaca oleracea* L.): A Critical Review. Environ. Manag..

[163] Zam W., Quispe C., Sharifi-Rad J., López M.D., Schoebitz M., Martorell M., Sharopov F., Fokou P.V.T., Mishra A.P., Chandran D. (2022). An Updated Review on The Properties of *Melissa officinalis* L.: Not Exclusively Anti-anxiety. Front. Biosci..

[164] Fuloria S., Mehta J., Chandel A., Sekar M., Rani N.N.I.M., Begum M.Y., Subramaniyan V., Chidambaram K., Thangavelu L., Nordin R. (2022). A Comprehensive Review on the Therapeutic Potential of *Curcuma longa* Linn. in Relation to its Major Active Constituent Curcumin. Front. Pharmacol..

[165] Shahane K., Kshirsagar M., Tambe S., Jain D., Rout S., Ferreira M.K.M., Mali S., Amin P., Srivastav P.P., Cruz J. (2023). An Updated Review on the Multifaceted Therapeutic Potential of *Calendula officinalis* L.. Pharmaceuticals.

[166] Andrade J.M., Faustino C., Garcia C., Ladeiras D., Reis C.P., Rijo P. (2018). *Rosmarinus officinalis* L.: An Update Review of Its Phytochemistry and Biological Activity. Future Sci. OA.

[167] Singh N., Sharma M.P., Gupta V.K. (2019). A Review on Pharmacological Aspects of Achyranthes aspera. Int. J. Pharmacogn. Phytochem. Res..

[168] Shaygannia E., Bahmani M., Zamanzad B., Rafieian-Kopaei M. (2016). A Review Study on *Punica granatum* L.. J. Evid. Based Complement. Altern. Med..

