# Supplementary material for: Innovative Bioactive Nanofibrous Materials Combining Medicinal and Aromatic Plant Extracts and Electrospinning Method
Source: Membranes (Basel). 2023 Oct 21;13(10):840. doi: 10.3390/membranes13100840 (PMC10608671; doi:10.3390/membranes13100840)
Supplement: Supplementary file 1 [file membranes-13-00840-s001.zip › membranes-2665346-supplementary.pdf]

Supplementary material
Manuscript ID: membranes-2665346

Table S1. The benefits of some plant extracts for health.

Plant	Part of plant	Beneficial properties	Reference
Achyrocline satureioides	Aerial	respiratory diseases, arthritis, stomach pain, to treat gastritis and ulcer, as antipyretic, wound healing, cholesterol lowering	[132] [133] [134]
Eupatorium viscidum	Aerial	acetylcholinesterase inhibition, modification of monoamines, antiamyloid aggregation effect and antioxidant activity	[135]
Ruprechtia apetala	Aerial	inhibition of tyrosinase monophenolase activity	[136]
Trichocline reptans	Aerial	cholinesterase inhibition	[137]
Zanthoxylum coco	Aerial	cholinesterase inhibition	[137]
Poncirus trifoliate	Fruit	mitigate allergic reactions, inflammation, edema, digestive complications, respiratory problems, spleen-related problems, liver complications, neuronal pain, hyperlipidemia, rheumatoid arthritis, cardiovascular problems, hernia, sinusitis, and insomnia	[138]
Treculia obovoidea	Twigs	antimicrobial activity	[139]
Angelica archangelica	Leaves and r oot	antioxidant activity, for digestive disorders, to enhance blood circulation	[140,141]
Cassia obtisufolia	Seed	neuroprotection, hepatoprotective effect, antimicrobial activity	[142,143,14 4]
Desmodium gangeticum	Root and leaf	tonics, febrifuge, digestive, antiemetic, astringent, anti-asthmatic, antidiarrhoeal and anti-inflammatory	[145, 146]
Salvia officinalis	Flowers, leaves	relief of pain, protecting the body against oxidative stress, free radical damages, angiogenesis, inflammation, bacterial and virus infection, anticancer and antiviral	[147, 148]
Moringa oleifera	Leaves, seeds, roots and flowers	antioxidant activity, wound healing	[149, 150, 151]

Ginkgo biloba	Leaves	neuroprotective, anticancer, cardioprotective, stress alleviating, and memory enhancing effects	[152]
Lavandula angustifolia	Flowers, buds and leaves	sedative and analgesic proper-ties, prevention of Alzheimer's disease, anticancer	[153]
Prunus armeniaca L	Fruit and seed	anticancer, gynecological diseases, rheumatic pain, headache, and skin hyperpigmentation, for skin diseases, ear inflammation, and tinnitus; treat asthma, productive cough, and fever	[154, 155]
Astragalus membranaceus	Root	anti-inflammatory, anticancer	[156]
Cordyceps sinensis	Fungus	increase longevity, anticancer, antioxidant properties, anti- diabetic, antiasthma, for treatment of cough and cold	[157]
Abelmoschus manihot	Flowers, seeds, stems, and leaves	treatment of chronic kidney disease, antidiabetic nephropathy, antioxidant, antiadipogenic, anti-inflammatory, analgesic, anticonvulsant, antidepressant, antiviral, antitumor, cardioprotective, antiplatelet, neuroprotective, immunomodulatory, and hepatoprotective activities	[158]
Vitis vinifera	Fruits, seeds, stems, skin and pomaces	chemoprotective properties against free radicals and oxidative stress, anti-inflammatory activity, and anticarcinogenic properties	[159]
Zingiber officinale	Root	antioxidant, anti-inflammatory, antimicrobial, and anticancer activities; for prevention of neurodegenerative diseases, cardiovascular diseases, obesity, diabetes mellitus, chemotherapy-induced nausea and emesis, for respiratory disorders	[160]
Garcinia lucida	Barks	chronic kidney disease and cardiovascular diseases treatment	[161]
Portulaca oleracea	Leaves	antioxidant activity, prevent cardiovascular disease and cancer, reducing atherosclerosis and inflammatory markers in the body, decelerate the progression of Parkinson's disease, improvement of blood lipid and glucose levels, treatment of diabetes and utilized as an anti-bacterial and anti-viral agent	[162]
Melissa Officinalis	Aerial	for treatment of gastrointestinal, cardiovascular, neurological, psychological disorders; antioxidant, anti-inflammatory,	[163]

		antispasmodic, antimicrobial, neuroprotective, nephroprotective, antinociceptive effects	
Curcuma longa	Roots	for treatment of liver obstruction and jaundice, for ulcers and inflammation; cough, cold, dental issues, indigestion, skin infections, blood purification, asthma, piles, bronchitis, tumor, wounds, and hepatic disorders	[164]
Calendula officinalis	Aerial	anti-inflammatory, anti-cancer, antihelminthic, antidiabetes, wound healing, hepatoprotective, and antioxidant activities	[165]
Rosmarinus officinalis	Aerial	antitumor, anti-inflammatory, analgesic, neurodegenerative, endocrinal, anti-infective and antioxidant; treat minor wounds, rashes, headache, dyspepsia, circulation problems	[166]
Achyranthes aspera	Aerial	antiperiodic, diuretic, purgative, laxative antiasthmatic, hepatoportective, anti-allergi; for treatment of pneumonia, diarrhea, dysentery, asthma, cough, dropsy, ulcers, piles, rheumatism, and skin diseases	[167]
Punica granatum	Fruits	anti-inflammatory, antioxidant and antibacterial activities; inhibitory effect on skin and breast cancers	[168]
